# Complex Dynamics of Noise-Perturbed Excitatory-Inhibitory Neural Networks With Intra-Correlative and Inter-Independent Connections

**DOI:** 10.3389/fphys.2022.915511

**Published:** 2022-06-24

**Authors:** Xiaoxiao Peng, Wei Lin

**Affiliations:** ^1^ Shanghai Center for Mathematical Sciences, School of Mathematical Sciences, and LMNS, Fudan University, Shanghai, China; ^2^ Research Institute of Intelligent Complex Systems and Center for Computational Systems Biology, Fudan University, Shanghai, China; ^3^ State Key Laboratory of Medical Neurobiology, MOE Frontiers Center for Brain Science, and Institutes of Brain Science, Fudan University, Shanghai, China

**Keywords:** complex dynamics, synchronization, neural network, excitatory, inhibitory, noise-perturbed, intra-correlative, inter-independent

## Abstract

Real neural system usually contains two types of neurons, i.e., excitatory neurons and inhibitory ones. Analytical and numerical interpretation of dynamics induced by different types of interactions among the neurons of two types is beneficial to understanding those physiological functions of the brain. Here, we articulate a model of noise-perturbed random neural networks containing both excitatory and inhibitory (E&I) populations. Particularly, both *intra-correlatively* and *inter-independently* connected neurons in two populations are taken into account, which is different from the most existing E&I models only considering the independently-connected neurons. By employing the typical mean-field theory, we obtain an equivalent system of two dimensions with an input of stationary Gaussian process. Investigating the stationary autocorrelation functions along the obtained system, we analytically find the parameters’ conditions under which the synchronized behaviors between the two populations are sufficiently emergent. Taking the maximal Lyapunov exponent as an index, we also find different critical values of the coupling strength coefficients for the chaotic excitatory neurons and for the chaotic inhibitory ones. Interestingly, we reveal that the noise is able to suppress chaotic dynamics of the random neural networks having neurons in two populations, while an appropriate amount of correlation coefficient in intra-coupling strengths can enhance chaos occurrence. Finally, we also detect a previously-reported phenomenon where the parameters region corresponds to neither linearly stable nor chaotic dynamics; however, the size of the region area crucially depends on the populations’ parameters.

## 1 Introduction

Collective behaviours induced by random interaction networks or/and external inputs are of omnipresent phenomena in many fields, such as signal processing (Boedecker et al., 2012; Wang et al., 2014), percolation (Chayes and Chayes, 1986; Transience, 2002), machine learning (Gelenbe, 1993; Jaeger and Haas, 2004; Gelenbe and Yin, 2016; Teng et al., 2018; Zhu et al., 2019), epidemic dynamics (Forgoston et al., 2009; Parshani et al., 2010; Dodds et al., 2013), and neuroscience (Shen and Wang, 2012; Giacomin et al., 2014; Aljadeff et al., 2015; Mastrogiuseppe and Ostojic, 2017). Elucidating the mechanisms that arouse such phenomena is beneficial to understanding and reproducing crucial functions of real-world systems.

Particularly, neuroscience is a realm where collective behaviours at different levels are ubiquitous, needing quantitative explorations by using the network models that take into account different types of perturbations, deterministic or stochastic. Somplinsky *et al.* articulated a general model describing autonomous, continuous, randomly coupled neural networks in a large scale and investigated how chaotic phase for this system is related crucially with the randomness of the network structure applying typical theory of mean-field and dimension reduction (Sompolinsky et al., 1988). Rajan *et al.* studied a random neural model with external periodic signals, where an appropriate amount of external signal is able to regularize the chaotic dynamics of the network model (Rajan et al., 2010). Schuecker *et al.* introduced the external signal in a manner of white noise to the neural network model, surprisingly finding a phase, neither chaotic nor linearly stable, maximizing the memory capacity of this network model (Schuecker et al., 2018). And some other computational neuroscientists investigated the influence of the random structures and stochastic perturbations positively or/and negatively on synchronization dynamics or computational behaviors in particularly coupled neural networks (Kim et al., 2020; Li et al., 2020; Pontes-Filho et al., 2020; Yang et al., 2022; Zhou et al., 2019; Leng and Aihara, 2020; Jiang and Lin, 2021).

Initially, the random neural network models studied in the literature include only one group/type of mutually connected neurons. However, physiological neurons usually have at least two types, excitatory neurons, and inhibitory ones. They are the elementary components in the cortex of the brain, where the output coupling strengths of excitatory (resp., inhibitory) neurons are non-negative (resp., non-positive) (Eccles, 1964; van Vreeswijk and Sompolinsky, 1996; Horn and Usher, 2013). Emerging evidences show that two types of neurons and their opposing coupling strengths are beneficial or disadvantageous to learning the patterns in cortical cell (van Vreeswijk and Sompolinsky, 1996), forming the working memory (Cheng et al., 2020; Yuan et al., 2021), and balancing the energy consumption and the neural coding (Wang and Wang, 2014; Wang et al., 2015). Thus, neural networks containing two types or populations of neurons, of practical significance, attract more and more attentions. Introducing two or even more populations of neurons in different types into the random network model naturally becomes an advancing mode for investigating how the types of neurons, together with the other physiological factors, influence the neurodynamics as well as the realization of the corresponding functions. For instance, the model that contains two types of neurons partially-connected was studied in (Brunel, 2000; Omri et al., 2015) and the model that contains neurons independently-connected was systematically investigated in (Hermann and Touboul, 2012; Kadmon and Sompolinsky, 2015).

**FIGURE 1:**
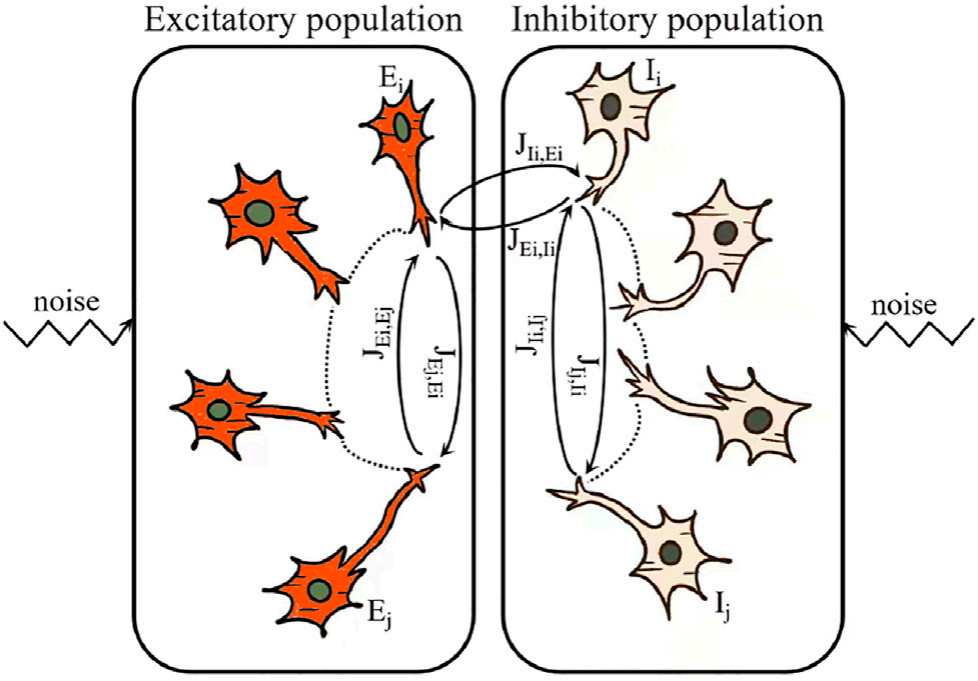
A sketch of the noise-perturbed excitatory-inhibitory neural networks. Here, based on the physiological evidences, *J*_*Ej*,*Ei*_ (resp., *J*_*Ii*,*Ij*_ and *J*_*Ij*,*Ii*_), the weights of the synapses connecting any pair of neurons in the same population *J*_*Ei*,*Ej*_ and are supposed to be correlated in our model, and *J*_*Ei*,*Ii*_ and *J*_*Ii*,*Ei*_ are supposed to be independent.

Experimental evidences further suggest that, for pairs of clustered cortical neurons, the bidirectional connections are hyper-reciprocal (Wang et al., 2006) and thus significantly correlated with each other (Litwin-Kumar and Doiron, 2012; Song et al., 2005). So, in this article, we intend to study the noise-perturbed excitatory-inhibitory random neural networks, in which the bidirectional synaptic weights between any pair of neurons in both type of populations are correlated, as showed sketchily in Figure 1. And we are to analytically investigate how such correlations affect the asymptotical collective behaviors of the coupled neurons in two populations. As such, our work could extend the results in (Daniel et al., 2017; Schuecker et al., 2018), where only one population is taken into account.

More precisely, this article is organized as follows. In Section 2, based on Somplinsky’s model of random neural networks, we introduce excitatory and inhibitory populations of intra-correlatively or inter-independently connected neurons, and further include the external perturbations of white noise into the model. Still in this section, we reduce this model of a large-scale into a two-dimensional system using the moment-generating functional and the saddle-point approximation (Eckmann and Ruelle, 1985; Gardiner, 2004; Helias and Dahmen, 2020). In Section 3, along with the reduced system, we study the autocorrelation function, which describes the population-averaged correlation of every state in the two-dimensional system at different time instants, and reveal how the external noise, the coupling strength, and the quantitative proportion of the neurons in two types together affect the autocorrelation function evolving in a long term. Also in this section, we specify some analytical conditions under which the whole system can synchronize as one population in a sense that the autocorrelation functions for the two populations of neurons behave in a consensus manner. Additionally in Section 4, we investigate the maximal Lyapunov exponents for the two populations, respectively, determining whether the system evolves chaotically (Eckmann and Ruelle, 1985), and thus seek numerically out the parameter regions where chaotic dynamics can be suppressed or enhanced. Still in this section, akin to the results obtained in (Schuecker et al., 2018), we numerically detect the parameters’ region where the system evolves in neither linearly stable nor chaotic manner, and also find that the size of such a region crucially depends on the statistical parameters of the two populations of the coupled neurons. We close this article by presenting some discussion and concluding remarks.

## 2 Model Formulation: Reduction From High-Dimension to Two-Dimension

To begin with, we introduce a neural network model constituting two interacted populations, either of which contains intra- and inter-interacted excitatory or inhibitory neurons. Such a model reads:

$$\left\{\begin{array}{c} \frac{dx_{Ei}}{dt}=-x_{Ei}+\overset{N_{E}}{\underset{j=1}{\sum }}J_{Ei,Ej}\phi \left(x_{Ej}\right)+\overset{N_{I}}{\underset{j=1}{\sum }}J_{Ei,Ij}\phi \left(x_{Ij}\right)+\sigma \xi_{Ei}\left(t\right),\\ \frac{dx_{Ii}}{dt}=-x_{Ii}+\overset{N_{E}}{\underset{j=1}{\sum }}J_{Ii,Ej}\phi \left(x_{Ej}\right)+\overset{N_{I}}{\underset{j=1}{\sum }}J_{Ii,Ij}\phi \left(x_{Ij}\right)+\sigma \xi_{Ii}\left(t\right), \end{array}\right.$$ (1)

where *x*_*Ei*_ and *x*_*Ii*_ represent the states of the excitatory neuron and the inhibitory one, respectively, and *ϕ*(⋅) stands for the transfer/activation function. Additionally, each *J*_*Ki*,*Lj*_ is the element of the random connection matrix specified below, and each *ξ*_*Ki*_(*t*), as the external perturbation, is supposed to be a white noise with an intensity *σ* and satisfying

$$\left\langle \xi_{Ki}\left(t\right)\xi_{Lj}\left(s\right)\right\rangle =\delta_{KL}\delta_{ij}\delta \left(t-s\right),$$

where *K*, *L* ∈ {*E*, *I*}, *i* = 1, 2, … , *N*_*K*_, *j* = 1, 2, … , *N*_*L*_, *δ*_*ij*_ is the Kronecker delta notation, and *δ*(*t* − *s*) is the Dirac delta function with *∫*_*R*_ *δ*(*x*)*f*(*x*)d*x* = *f*(0).

On one hand, based on the experimental evidences (Song et al., 2005; Wang et al., 2006; Litwin-Kumar and Doiron, 2012), we suppose that the strength of the intra-coupling between any two of the neurons in the same population is *correlated*, and further that the mean strength of these couplings does not vanish. Specifically, we suppose the couplings to obey the Gaussian normal distributions as

$$\left(\begin{array}{c} J_{Ei,Ej}\\ J_{Ej,Ei} \end{array}\right)∼N\left(M_{E},A_{E}\right),\;\;\left(\begin{array}{c} J_{Ii,Ij}\\ J_{Ij,Ii} \end{array}\right)∼N\left(M_{I},A_{I}\right),$$

in which

$$\begin{array}{ll} M_{E} & ={\left(\begin{array}{cc} m_{EE}/N_{E} & m_{EE}/N_{E} \end{array}\right)}^{⊤},\;M_{I}={\left(\begin{array}{cc} m_{II}/N_{I} & m_{II}/N_{I} \end{array}\right)}^{⊤},\\ A_{E} & =g^{2}\left(\begin{array}{cc} \frac{1}{N} & \frac{\eta_{E}}{N}\\ \frac{\eta_{E}}{N} & \frac{1}{N} \end{array}\right),\;A_{I}=g^{2}\left(\begin{array}{cc} \frac{1}{N} & \frac{\eta_{I}}{N}\\ \frac{\eta_{I}}{N} & \frac{1}{N} \end{array}\right). \end{array}$$

Here, *g* is the gain parameter modulating the coupling strength of the overall network, each *N*_*E*_ (resp., *N*_*I*_) stands for the number of neurons in the excitatory (resp., inhibitory) population with *N* = *N*_*E*_ + *N*_*I*_, each *η*_*E*_ (resp., *η*_*I*_) describes the correlation between *J*_*Ei*,*Ej*_ and *J*_*Ej*,*Ei*_ (resp., *J*_*Ii*,*Ij*_ and *J*_*Ij*,*Ii*_), two weights of the synapses connecting any pair of the neurons in the same excitatory (resp., inhibitory) population, and *m*_*EE*_ (resp., *m*_*II*_) characterizes the mean coupling strength among the excitatory (resp., inhibitory) population.

On the other hand, as usual, we assume that the strength of the inter-coupling between any pair of neurons, respectively, from the two populations is independent of the other strength, and further that they obey the following distributions:

$$J_{Ei,Ij}∼N\left(m_{EI}/N_{I},g^{2}/N\right),\;\;J_{Ii,Ej}∼N\left(m_{IE}/N_{E},g^{2}/N\right),$$

where *m*_*EI*_ (resp., *m*_*IE*_) is the mean coupling strength from the neurons in the inhibitory (resp., excitatory) population to the ones in the excitatory (resp., inhibitory) population. In the following investigations, we assume that *N*_*E*_/*N*_*I*_, the proportion, does not change as *N* grows. Clearly, there are two kinds of randomnesses simultaneously in this model: *J*_*Ki*,*Lj*_, the random coupling strengths, and *ξ*_*Ki*_, the external perturbations of white noise. In fact, such a model is also suitable for describing coherent or incoherent phenomena that are produced by any system of two populations in addition to the neural populations that are discussed in this article.

Since the coupling matrices used in system (1) are randomly generated during the numerical realizations, simulated dynamics of this system are different almost surely for different selected matrices. To obtain deep, analytical insights to the asymptotic properties of this system as the number of populations *N* goes to the infinity, we utilize a mean-field technique. Specifically, applying the Martin–Siggia–Rose–de Dominicis–Janssen path integral formula (Martin et al., 1973; De Dominicis, 1976; Janssen, 1976; Chow and Buice, 2015), we transform system (1), stochastic differential equations, into an Itô integral form, and derive the following moment-generating functional:

$$Z\left[l\right]\left(J\right)=\int Dx\int D\tilde{x}\;\exp\left\{S\left[x,\tilde{x}\right]-{\tilde{x}}^{⊤}J\phi \left(x\right)+l^{⊤}x\right\}.$$ (2)

Here, for ease of reading, we include the tedious calculations for obtaining this functional into Supplementary Appendix SA. The notations in (2) are illustrated as follows:

$$S\left[x,\tilde{x}\right]={\tilde{x}}^{T}\left(\partial_{t}+1\right)x+\frac{1}{2}\sigma^{2}{\tilde{x}}^{⊤}x$$

and $x≔{\left(\begin{array}{cc} x_{E} & x_{I} \end{array}\right)}^{⊤}$ represents the trajectories of the neurons with

$$x_{E}≔{\left(x_{Ei}\left(t\right),\;t\in R\right)}_{i=1}^{N_{E}},\;x_{I}≔{\left(x_{Ii}\left(t\right),\;t\in R\right)}_{i=1}^{N_{I}}.$$

Moreover, after an inverse Fourier transformation of the Dirac delta function, $\tilde{x}≔{\left(\begin{array}{cc} {\tilde{x}}_{E} & {\tilde{x}}_{I} \end{array}\right)}^{⊤}$ represents the response field with

$${\tilde{x}}_{E}≔{\left({\tilde{x}}_{Ei}\left(t\right),\;t\in R\right)}_{i=1}^{N_{E}},\;{\tilde{x}}_{I}≔{\left({\tilde{x}}_{Ii}\left(t\right),\;t\in R\right)}_{i=1}^{N_{I}},$$

and $l≔{\left(\begin{array}{cc} l_{E} & l_{I} \end{array}\right)}^{⊤}$ represents the source field where

$$l_{E}≔{\left(l_{Ei}\left(t\right),\;t\in R\right)}_{i=1}^{N_{E}},\;l_{I}≔{\left(l_{Ii}\left(t\right),\;t\in R\right)}_{i=1}^{N_{I}}.$$

The formula

$$l^{⊤}x≔\overset{\;}{\underset{K\in \left\{E,I\right\}}{\sum }}\overset{N_{K}}{\underset{i=1}{\sum }}\int_{R}l_{K,i}\left(t\right)x_{K,i}\left(t\right)dt$$

denotes the inner product in time and two populations of neurons. In addition, the measure here is denoted formally as

$$\int Dx=\underset{M\to \infty }{\lim}\overset{\;}{\underset{K\in \left\{E,I\right\}}{\prod }}\overset{N_{K}}{\underset{i=1}{\prod }}\overset{M}{\underset{t=1}{\prod }}\int_{-\infty }^{+\infty }dx_{Ki}^{t}$$

and

$$\int D\tilde{x}=\underset{M\to \infty }{\lim}\overset{\;}{\underset{K\in \left\{E,I\right\}}{\prod }}\overset{N_{K}}{\underset{i=1}{\prod }}\overset{M}{\underset{t=1}{\prod }}\int_{-i\infty }^{+i\infty }\frac{d{\tilde{x}}_{Ki}^{t}}{2\pi i}.$$

Finally, ***J*** is an *N* × *N* matrix partitioned as

$$J=\left(\begin{array}{cc} J_{EE} & J_{EI}\\ J_{IE} & J_{II} \end{array}\right),$$

where $J_{KL}={\left(J_{Ki,Lj}\right)}_{N_{K}\times N_{L}}$ .

With the moment-generating function *Z* obtained in (2), we average it over the random coupling matrix ***J***, perform a saddle-point approximation method, and reduce the model into a two-dimensional system, which reads

$$\left\{\begin{array}{cc} \frac{dx_{E}}{dt}\; & =-x_{E}+\gamma_{E}\left(t\right)+\sigma \xi_{E}\left(t\right)+m_{EE}\langle \phi \left(x_{E}\left(t\right)\right)\rangle +m_{EI}\langle \phi \left(x_{I}\left(t\right)\right)\rangle ,\\ \frac{dx_{I}}{dt}\; & =-x_{I}+\gamma_{I}\left(t\right)+\sigma \xi_{I}\left(t\right)+m_{IE}\langle \phi \left(x_{E}\left(t\right)\right)\rangle +m_{II}\langle \phi \left(x_{I}\left(t\right)\right)\rangle . \end{array}\right.$$ (3)

Here, for a concise expression, we still include the detailed arguments into Supplementary Appendix SB. The notations used in (3) are defined as follows: *ξ*_*K*_(*t*) with *K* ∈ {*E*, *I*} are mutually independent white noises, and *γ*_*K*_(*t*) with *K* ∈ {*E*, *I*} are mutually independent stationary Gaussian processes with their means as zeros and their correlations satisfying

$$\begin{array}{ccc} \langle \gamma_{E}\left(t\right)\gamma_{E}\left(t^{\prime }\right)\rangle & = & \frac{g^{2}}{N}\left[N_{E}\left(1+\eta_{E}\right)\langle \phi \left(x_{E}\left(t\right)\right)\phi \left(x_{E}\left(t^{\prime }\right)\right)\rangle +N_{I}\langle \phi \left(x_{I}\left(t\right)\right)\phi \left(x_{I}\left(t^{\prime }\right)\right)\rangle \right],\\ \langle \gamma_{I}\left(t\right)\gamma_{I}\left(t^{\prime }\right)\rangle & = & \frac{g^{2}}{N}\left[N_{E}\langle \phi \left(x_{E}\left(t\right)\right)\phi \left(x_{E}\left(t^{\prime }\right)\right)\rangle +N_{I}\left(1+\eta_{I}\right)\langle \phi \left(x_{I}\left(t\right)\right)\phi \left(x_{I}\left(t^{\prime }\right)\right)\rangle \right]. \end{array}$$

## 3 Dynamics of Autocorrelation Functions

Here, we investigate the dynamics of the autocorrelation functions for the reduced system (3). To this end, letting the left hand side of system (3) equal to zeros yields a stationary solution satisfying

$$\left\{\begin{array}{cc} \langle x_{E}\rangle \; & =m_{EE}\langle \phi \left(x_{E}\right)\rangle +m_{EI}\langle \phi \left(x_{I}\right)\rangle ,\\ \langle x_{I}\rangle \; & =m_{IE}\langle \phi \left(x_{E}\right)\rangle +m_{II}\langle \phi \left(x_{I}\right)\rangle . \end{array}\right.$$ (4)

For an odd transfer function, we immediately obtain that ⟨*x*_*E*_⟩ = ⟨*x*_*I*_⟩ = 0 is a consistent solution. Using the error dynamics as

$$\left\{\begin{array}{cc} \delta x_{E}\left(t\right)\; & =x_{E}\left(t\right)-\langle x_{E}\rangle ,\\ \delta x_{I}\left(t\right)\; & =x_{I}\left(t\right)-\langle x_{I}\rangle , \end{array}\right.$$

and assuming these dynamics as stationary Gaussian processes, we define the autocorrelation functions as

$$C_{E}\left(\tau \right)≔\langle \delta x_{E}\left(t\right)\delta x_{E}\left(t+\tau \right)\rangle ,\;\;C_{I}\left(\tau \right)≔\langle \delta x_{I}\left(t\right)\delta x_{I}\left(t+\tau \right)\rangle ,$$

where, physically, these functions represent the population-averaged cross-correlations between the dynamical quantities deviating from the equilibrium states at *t* and *t* + *τ*, two different time instants. For a given difference *τ* between the time instants, the larger the value of the autocorrelation function, the more correlative manner the deviating dynamics behave in. As will be numerically displayed in the following investigation, with the increase of the difference *τ*, the value sometimes drops down but sometimes exhibits a non-monotonic tendency.

Further define the variances by *c*_*E*0_≔*C*_*E*_(0) and *c*_*I*0_≔*C*_*I*_(0), and define *c*_*E∞*_≔ lim_*τ*→*∞*_ *C*_*E*_(*τ*) and *c*_*I∞*_≔ lim_*τ*→*∞*_ *C*_*I*_(*τ*). Through implementing the arguments performed in Supplementary Appendix SC, we get two population-averaged autocorrelation functions obeying the following second-order equations:

$$\left\{\begin{array}{cc} \frac{d^{2}C_{E}}{d\tau^{2}} & =C_{E}-\frac{g^{2}}{N}\left[N_{E}\left(\eta_{E}+1\right)\langle \phi \left(x_{E}\left(t\right)\right)\phi \left(x_{E}\left(t+\tau \right)\right)\rangle +N_{I}\langle \phi \left(x_{I}\left(t\right)\right)\phi \left(x_{I}\left(t+\tau \right)\right)\rangle \right]\\ & -\sigma^{2}\delta \left(\tau \right),\\ \frac{d^{2}C_{I}}{d\tau^{2}} & =C_{I}-\frac{g^{2}}{N}\left[N_{E}\langle \phi \left(x_{E}\left(t\right)\right)\phi \left(x_{E}\left(t+\tau \right)\right)\rangle +N_{I}\left(\eta_{I}+1\right)\langle \phi \left(x_{I}\left(t\right)\right)\phi \left(x_{I}\left(t+\tau \right)\right)\rangle \right]\\ & -\sigma^{2}\delta \left(\tau \right). \end{array}\right.$$ (5)

Notice that each element of system (3) is a Gaussian process and that

$$\begin{array}{ll} \left\langle \phi \left(x_{K}\left(t\right)\right)\phi \left(x_{K}\left(t+\tau \right)\right)\right\rangle & =\frac{1}{2\pi \sqrt{\left|A\right|}}\iint_{R^{2}}\phi \left(z_{1}+\left\langle x_{K}\right\rangle \right)\phi \left(z_{2}+\left\langle x_{K}\right\rangle \right)\\ & \;\exp\left(-\frac{1}{2}z^{T}A^{-1}z\right)dz_{1}dz_{2}, \end{array}$$ (6)

where

$$A=\left(\begin{array}{cc} c_{K0} & C_{K}\left(\tau \right)\\ C_{K}\left(\tau \right) & c_{K0} \end{array}\right),\;\;\;K\in \left\{E,I\right\}.$$

We thus introduce the notation $f_{\phi \left(\cdot +\left\langle x_{K}\right\rangle \right)}\left(C_{K}\left(\tau \right),c_{K0}\right)≔\left\langle \phi \left(x_{K}\left(t\right)\right)\phi \left(x_{K}\left(t+\tau \right)\right)\right\rangle$ to represent the function with respect to the autocorrelation and the variance for each *K*.

In what follows, we analytically and numerically depict how the dynamics of autocorrelation functions are affected by external noise, the coupling strength, and the quantitative proportion of the neurons in two populations. Also we establish conditions under which these two populations of neurons can be synchronized. More precisely, the analytical results are summarized into the following propositions and the numerical results are illustrated after the propositions. The detailed arguments and computations for validating these propositions are included in Supplementary Appendix SD.

**Proposition III.1** Define the potentials of the population-averaged motion for the two types of neurons by

$$\left\{\begin{array}{cc} V_{E}\left(C_{E},C_{I};c_{E0},c_{I0}\right)\; & =-\frac{1}{2}C_{E}^{2}+\frac{g^{2}N_{E}\left(1+\eta_{E}\right)}{N}f_{\rho_{E}}\left(C_{E},c_{E0}\right)+\frac{g^{2}N_{I}}{N}f_{\rho_{I}}\left(C_{I},c_{I0}\right),\\ V_{I}\left(C_{E},C_{I};c_{E0},c_{I0}\right)\; & =-\frac{1}{2}C_{I}^{2}+\frac{g^{2}N_{E}}{N}f_{\rho_{E}}\left(C_{E},c_{E0}\right)+\frac{g^{2}N_{I}\left(1+\eta_{I}\right)}{N}f_{\rho_{I}}\left(C_{I},c_{I0}\right), \end{array}\right.$$ (7)

where

$$\rho_{K}\left(x\right)=\int_{0}^{x}\phi \left(y+\left\langle x_{K}\right\rangle \right)dy,\;K\in \left\{E,I\right\},$$

and assume that the external white noise contribute to the initial kinetic energy of all the neurons. Then, the autocorrelation functions satisfy

$$\left\{\begin{array}{cc} \frac{1}{2}{C_{E}^{\prime }}^{2}+V_{E}\left(C_{E},C_{I};c_{E0},c_{I0}\right)\; & =const,\\ \frac{1}{2}{C_{I}^{\prime }}^{2}+V_{I}\left(C_{E},C_{I};c_{E0},c_{I0}\right)\; & =const, \end{array}\right.$$ (8)

and the consistent solutions of *c_K∞_* and *c_K*0*_* satisfy

$$\left\{\begin{array}{c} \frac{\sigma^{4}}{8}+V_{E}\left(c_{E0},c_{I0};c_{E0},c_{I0}\right)=V_{E}\left(c_{E\infty },c_{I\infty };c_{E0},c_{I0}\right),\\ \frac{\sigma^{4}}{8}+V_{I}\left(c_{E0},c_{I0};c_{E0},c_{I0}\right)=V_{I}\left(c_{E\infty },c_{I\infty };c_{E0},c_{I0}\right). \end{array}\right.$$ (9)

Additionally, define the powers of the motions (i.e., the derivatives of *− V_K_* as defined above) by

$$\left\{\begin{array}{c} W_{E}\left(C_{E},C_{I};c_{E0},c_{I0}\right)=C_{E}-\frac{g^{2}N_{E}\left(1+\eta_{E}\right)}{N}f_{\phi \left(\cdot +\langle x_{E}\rangle \right)}\left(C_{E},c_{E0}\right)-\frac{g^{2}N_{I}}{N}f_{\phi \left(\cdot +\langle x_{I}\rangle \right)}\left(C_{I},c_{I0}\right),\\ W_{I}\left(C_{E},C_{I};c_{E0},c_{I0}\right)=C_{I}-\frac{g^{2}N_{E}}{N}f_{\phi \left(\cdot +\langle x_{E}\rangle \right)}\left(C_{E},c_{E0}\right)-\frac{g^{2}N_{I}\left(1+\eta_{I}\right)}{N}f_{\phi \left(\cdot +\langle x_{I}\rangle \right)}\left(C_{I},c_{I0}\right), \end{array}\right.$$ (10)

and suppose that these powers dissipate in a long timescale, that is,

$$W_{E}\left(c_{E\infty },c_{I\infty };c_{E0},c_{I0}\right)=W_{I}\left(c_{E\infty },c_{I\infty };c_{E0},c_{I0}\right)=0.$$ (11)

Then, the two mean states *⟨x_K_⟩*, the two self-variances *c_K0_*, and the two correlations in a long term *c_K∞_* for the two populations are analytically existent provided that the equations specified in (4), (9), and (11) are consistent.

Particularly, Eq. 8 is actually the energy conservation equation with the potentials *V*_*E*,*I*_. Using Eq. 9 yields that the intensity *σ* of the white noise contributes to the initial kinetic energy $\frac{1}{2}{C_{E}^{\prime }}^{2}\left(0\right)=\frac{1}{2}{C_{I}^{\prime }}^{2}\left(0\right)=\frac{\sigma^{4}}{8}$ with the initial velocity $C_{E}^{\prime }\left(0\right)=C_{I}^{\prime }\left(0\right)=\frac{\sigma^{2}}{2}$ . Thus, the self-variance *c*_*K*0_ can admit a higher value than *c*_*K∞*_.

The following proposition further describes the specific forms of the solutions to the consistent equations in 4, 9, 11 under particular conditions.

**Proposition III.2** Suppose that all the assumptions set in Proposition III. 1 are all valid. Then, we have the following results.

1) If the transfer function is odd, the two correlations in a long term satisfy *c*_*E∞*_ = *c*_*I∞*_ = 0. If *σ* = 0 is further fulfilled, the two self-variances satisfy *c*_*E*0_ = *c*_*I*0_ = 0.
2) If one of the following three conditions is satisfied:
  i) all the coupling strengths are independent with each other, i.e., *η*_*E*_ = *η*_*I*_ = 0.
  ii) the transfer function is odd and *N_E_η_E_ = N_I_η_I_*, and.
  iii) the mean states satisfy *⟨x_E_⟩ = ⟨x_I_⟩* and the population sizes with the coupling strengths fulfilling *N_E_η_E_ = N_I_η_I_*,

then we have *C_E_(τ) = C_I_(τ)*. This implies that the two populations becomes synchronized in a sense that the two stationary autocorrelation functions behave in the same manner.

**FIGURE 2:**
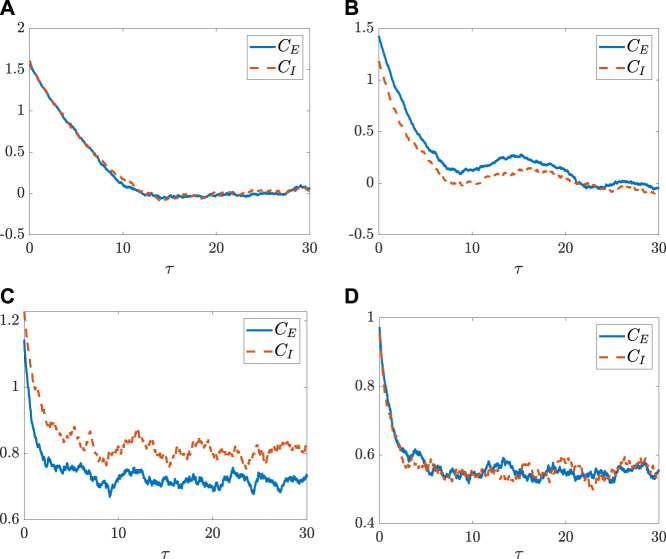
The autocorrelation functions *C*_*E*,*I*_(*τ*) change with an increase of the time difference *τ* when the system goes over the time duration from *t* = 0 to 100. **(A)** The parameters are set as *η*_*E*_ *N*_*E*_ = *η*_*I*_ *N*_*I*_ = 500 with *N*_*E*_ = 1,500, *N*_*I*_ = 1,000, *m*_*EE*_ = 1, *m*_*IE*_ = 0.75 *m*_*EI*_ = −0.25, *m*_*II*_ = −0.5, *g* = 1.4, *σ* = 0.8, $\eta_{E}=\frac{1}{3}$ , and $\eta_{I}=\frac{1}{2}$ . The transfer function is set as *ϕ*(*x*) = arctanx. **(B)** All the configurations are the same as those set in **(A)** except for *η*_*I*_ = 0. **(C)** All the configurations are the same as those set in **(A)** except for the transfer function set as *ϕ*(*x*) = max(arctan*x*, 0) and the parameters changed as *m*_*IE*_ = 75 and *m*_*II*_ = −50. **(D)** All the configurations are the same as those set in **(C)** except for *η*_*E*_ = *η*_*I*_ = 0. Here, only one realization for each functions are numerically presented.

The following numerical simulations further demonstrate the above analytical findings on the synchronization of the two populations of the neurons. More specifically, for the case where the transfer function is set as an inverse of the tangent function and *η*_*E*_ *N*_*E*_ = *η*_*I*_ *N*_*I*_, the autocorrelation functions of the two populations are equal all the time according to Proposition III. 2. The fluctuating tendencies of the autocorrelation functions *C*_*E*,*I*_(*τ*) plotted in Figure 2A validate such a case, although this figure presents only one realization for each *C*_*K*_(*τ*). Thus, the potentials *V*_*E*,*I*_ for the two populations are all the same along the time, which implies the occurrence of the synchronized dynamics for the two populations. As seen in Figure 2B (resp., Figure 2C), for the case where *η*_*E*_ *N*_*E*_ = *η*_*I*_ *N*_*I*_ (resp., ⟨*x*_*E*_⟩ = ⟨*x*_*I*_⟩) is not satisfied, the dynamics of autocorrelation function for the two populations become different completely. As shown by one realization of each *C*_*K*_(*τ*) in Figure 2D, as the coupling correlation coefficient *η* vanishes, the dynamics behave in a similar manner again. Additionally, as indicated by Proposition III.2, for the transfer function as an inverse of the tangent function, we have *c*_*E∞*_ = *c*_*I∞*_ = 0 (see Figures 2A,B); however, for the transfer function as a non-odd function, their autocorrelation functions in a long term may not ever vanish (see Figures 2C,D).

## 4 Route to Chaos

Since chaos emergent in neural network models is often regarded as a crucial dynamical behavior beneficial to memory storage and association capacity (Lin and Chen, 2009; Haykin et al., 2006; Toyoizumi and Abbott, 2011; Schuecker et al., 2018), it is of great interest to investigate the route of generating chaos in the above system (3) of two populations of neurons. To this end, we calculate the maximal Lyapunov exponent, which quantifies the asymptotic growth rate of the system dynamics for given initial perturbations (Cencini et al., 2010). More precisely, for the two dynamics $x_{K}^{1}\left(t\right)$ and $x_{K}^{2}\left(t\right)$ in one of the populations and with the same realization of the coupling matrix ***J*** and the white noise ***ξ***, the maximal Lyapunov exponent (MLE) is defined by

$$\lambda_{\text{max},K}≔\underset{t\to \infty }{\lim}\underset{\|x_{K}^{1}\left(0\right)-x_{K}^{2}\left(0\right)\|\to 0}{\lim}\frac{1}{t}\ln\frac{\left\|x_{K}^{1}\left(t\right)-x_{K}^{2}\left(t\right)\right\|}{\left\|x_{K}^{1}\left(0\right)-x_{K}^{2}\left(0\right)\right\|},$$

where ‖ ⋅‖ stands for the Euclidean-norm, the number in the superscript corresponds to the different group of the initial values, and the notation (*K* ∈ {*E*, *I*}) in the subscript corresponds to the different population of the neurons. Still for the same realization of the coupling matrix and the white noise, denote, component-wisely, by $x_{Ei}^{\alpha }$ (resp., $x_{Ii}^{\alpha }$ ) the state of the *i*-th neuron in the excitatory (resp., inhibitory) population with the *α*-th group of initial values. Here, *α* = 1, 2, *K* ∈ {*E*, *I*} and *i* = 1, 2, … , *N*_*K*_. Actually, the MLE quantifies how the sensitivity of a system depends on its initial values. We, following the idea presented in Ref. (Derrida and Pomeau, 1986), calculate the MLEs on the two dynamics with only initial values different. Define the distance between these two dynamics, in either population but with different initial values, by

$$d_{K}\left(t\right)≔\frac{1}{N_{K}}\overset{N_{K}}{\underset{i=1}{\sum }}{\left[x_{Ki}^{1}\left(t\right)-x_{Ki}^{2}\left(t\right)\right]}^{2}=\frac{1}{N_{K}}\overset{N_{K}}{\underset{i=1}{\sum }}{\left[\delta x_{Ki}^{1}\left(t\right)-\delta x_{Ki}^{2}\left(t\right)\right]}^{2},\;\;K\in \left\{E,I\right\}.$$

Clearly, if *λ*_max,*E*_ > 0 (resp., *λ*_max,*I*_ > 0), that is, the distance between the two dynamics in the excitatory (resp., inhibitory) population grows exponentially with arbitrarily-closed initial values, the dynamics of this population become chaotic. In order to show the conditions under which the chaos occurs, we evaluate *d*_*K*_(*t*) as specified above. Thus, direct computation and approximation yield:

$$d_{K}\left(t\right)\approx C_{K}^{11}\left(t,t\right)+C_{K}^{22}\left(t,t\right)-C_{K}^{12}\left(t,t\right)-C_{K}^{21}\left(t,t\right),$$

where

$$C_{K}^{\alpha \beta }\left(s,t\right)≔\left\langle \delta x_{K}^{\alpha }\left(s\right)\delta x_{K}^{\beta }\left(t\right)\right\rangle ,\;\;K\in \left\{E,I\right\},\;\alpha ,\beta =1,2.$$

To study the dynamics of *d*_*K*_(*t*) more explicitly, we define

$$d_{K}\left(t,t^{\prime }\right)≔C_{K}^{11}\left(t,t^{\prime }\right)+C_{K}^{22}\left(t,t^{\prime }\right)-C_{K}^{12}\left(t,t^{\prime }\right)-C_{K}^{21}\left(t,t^{\prime }\right),$$

where *d*_*K*_(*t*, *t*) = *d*_*K*_(*t*), each $C_{K}^{\alpha \alpha }$ with *α* = 1, 2 is the autocorrelation function of the dynamics starting from a given group of initial values, and $C_{K}^{\alpha \beta }$ is the cross-correlation function of the two dynamics with $C_{K}^{12}=C_{K}^{21}$ . Then, we focus on the collective behaviours of the dynamics starting from different groups of initial values. Particularly, we apply the moment-generating functional, akin to the arguments performed in Supplementary Appendix SA, and obtain

$$Z\left[l^{1},l^{2}\right]\left(J\right)=\overset{2}{\underset{\alpha =1}{\prod }}\int Dx^{\alpha }\int D{\tilde{x}}^{\alpha }\left(\exp\left\{S\left[x^{\alpha },{\tilde{x}}^{\alpha }\right]-{\tilde{x}}^{\alpha ⊤}J\phi \left(x^{\alpha }\right)+l^{\alpha ⊤}x^{\alpha }\right\}\right)\exp\left(\sigma^{2}{\tilde{x}}^{1⊤}{\tilde{x}}^{2}\right).$$

Analogously, averaging it over the random coupling matrix and making a saddle-point approximation (see Supplementary Appendix SE for details) give equivalent dynamical equations as follows.

$$\left\{\begin{array}{cc} \frac{dx_{E}^{\alpha }}{dt}\; & =-x_{E}^{\alpha }+\gamma_{E}^{\alpha }\left(t\right)+\sigma \xi_{E}^{\alpha }\left(t\right)+m_{EE}\langle \phi \left(x_{E}^{\alpha }\left(t\right)\right)\rangle +m_{EI}\langle \phi \left(x_{I}^{\alpha }\left(t\right)\right)\rangle ,\\ \frac{dx_{I}^{\alpha }}{dt}\; & =-x_{I}^{\alpha }+\gamma_{I}^{\alpha }\left(t\right)+\sigma \xi_{I}^{\alpha }\left(t\right)+m_{IE}\langle \phi \left(x_{E}^{\alpha }\left(t\right)\right)\rangle +m_{II}\langle \phi \left(x_{I}^{\alpha }\left(t\right)\right)\rangle , \end{array}\right.$$

where *α* = 1, 2, $\xi_{i}^{\alpha }\left(t\right)$ is the independent white noise and $\gamma_{i}^{\alpha }\left(t\right)$ is the stationary Gaussian process with its mean as zero and its correlation satisfying

$$\begin{array}{ccc} \langle \gamma_{E}^{\alpha }\left(t\right)\gamma_{E}^{\beta }\left(t^{\prime }\right)\rangle & =\frac{g^{2}}{N}\left[N_{E}\left(1+\eta_{E}\right)\langle \phi \left(x_{E}^{\alpha }\left(t\right)\right)\phi \left(x_{E}^{\beta }\left(t^{\prime }\right)\right)\rangle +N_{I}\langle \phi \left(x_{I}^{\alpha }\left(t\right)\right)\phi \left(x_{I}^{\beta }\left(t^{\prime }\right)\right)\rangle \right], & \\ \langle \gamma_{I}^{\alpha }\left(t\right)\gamma_{I}^{\beta }\left(t^{\prime }\right)\rangle & =\frac{g^{2}}{N}\left[N_{E}\langle \phi \left(x_{E}^{\alpha }\left(t\right)\right)\phi \left(x_{E}^{\beta }\left(t^{\prime }\right)\right)\rangle +N_{I}\left(1+\eta_{I}\right)\langle \phi \left(x_{I}^{\alpha }\left(t\right)\right)\phi \left(x_{I}^{\beta }\left(t^{\prime }\right)\right)\rangle \right] & \end{array}$$

with *α*, *β* = 1, 2. Now, inspired by the ansatz proposed in (Schuecker et al., 2018), we expand the cross-correlation functions as

$$C_{K}^{12}\left(t,t^{\prime }\right)=C_{K}\left(t-t^{\prime }\right)+\epsilon G_{K}\left(t,t^{\prime }\right),$$ (12)

where $C_{K}\left(t-t^{\prime }\right)=C_{K}^{11}\left(t,t^{\prime }\right)=C_{K}^{22}\left(t,t^{\prime }\right)$ and *K* ∈ {*E*, *I*}. Hence, we get *d*_*K*_(*t*) = −2*ϵG*_*K*_(*t*, *t*) and the dynamical equations for the error dynamics of the cross-correlation functions from the autocorrelation functions as follows:

$$\begin{array}{cc} \left(\partial_{t^{\prime }}+1\right)\left(\partial_{t}+1\right)G_{K}\left(t,t^{\prime }\right) & =\frac{g^{2}}{N}\left[N_{E}\left(1+\delta_{KE}\eta_{E}\right)f_{\phi^{\prime }\left(\cdot +\langle x_{E}\rangle \right)}\left(C_{E}\left(t-t^{\prime }\right),c_{E0}\right)G_{E}\left(t,t^{\prime }\right)\right.\\ & \left.+N_{I}\left(1+\delta_{KI}\eta_{I}\right)f_{\phi^{\prime }\left(\cdot +\langle x_{I}\rangle \right)}\left(C_{I}\left(t-t^{\prime }\right),c_{I0}\right)G_{I}\left(t,t^{\prime }\right)\right] \end{array}$$ (13)

with *K* ∈ {*E*, *I*}. Refer to Supplementary Appendix SF for the detailed arguments on obtaining the above equations. Based on these equations, we can obtain the conditions under which the two populations of the neurons have the same asymptotic behaviours of the MLEs, and specify the critical value of the coupling strength coefficient *g* where the two populations simultaneously behave in a chaotic manner. We summarize the main conclusion into the following proposition and include the detailed arguments into Supplementary Appendix SG.

Proposition IV.1 Suppose that either one of the conditions assumed in conclusion (2) of Proposition III.2 is satisfied. Then, we have that *G_E_(t, t′) = G_I_(t, t′)* and that the MLEs are the same for the two populations. Particularly, *g_K,c_*, the critical value for generating chaos in the two populations, are the same and satisfy

$$c_{K0}-g_{K,c}^{2}\left(1+\frac{N_{K}\eta_{K}}{N}\right)f_{\phi }\left(c_{K0},c_{K0}\right)=0,\;\;K\in \left\{E,I\right\}.$$ (14)

**FIGURE 3:**
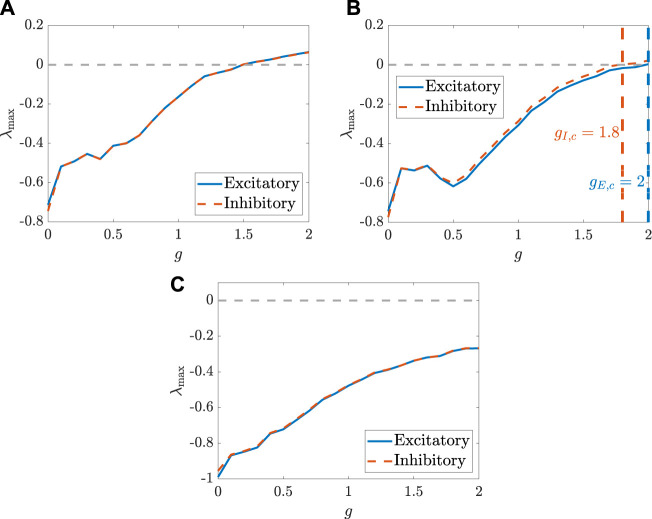
The MLEs, respectively, for the two populations, change with the coupling strength coefficient *g*. **(A)** The parameters are set as: *N*_*E*_ = 1,500, *N*_*I*_ = 1,000, *m*_*EE*_ = 1, *m*_*IE*_ = 0.75 *m*_*EI*_ = −0.25, *m*_*II*_ = −0.5, *σ* = 0.8, $\eta_{E}=\frac{1}{3}$ , and $\eta_{I}=\frac{1}{2}$ . The transfer function is selected as *ϕ*(*x*) = arctan*x*. **(B)** All the configurations are used in the same manner as those used in **(A)** except for *η*_*E*_ = −1. **(C)** All the configurations are used in the same manner as those used in **(A)** except for the parameters set as *m*_*EI*_ = −25 and *m*_*II*_ = −50 and the transfer function set as *ϕ*(*x*) = max(arctan*x*, 0).

**FIGURE 4:**
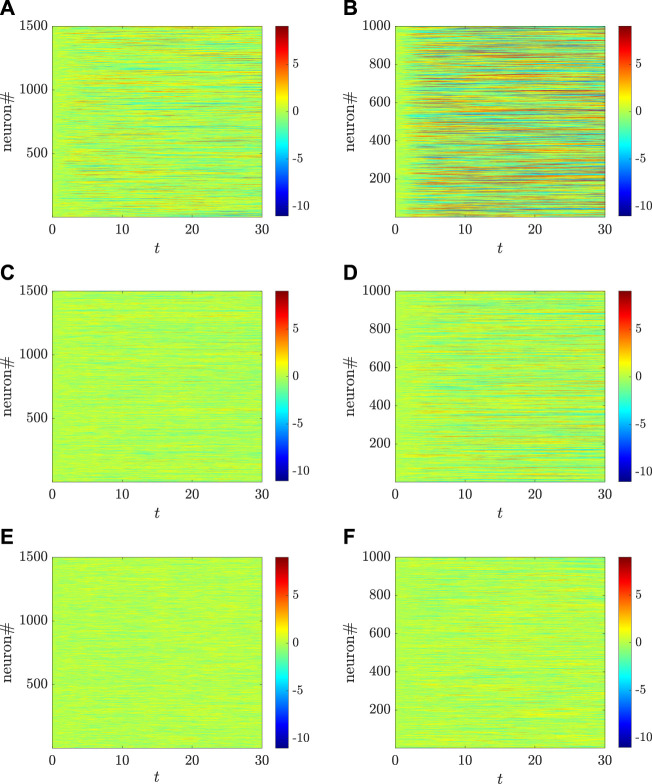
Different dynamical behaviors, respectively, for the excitatory **(A,C,E)** and the inhibitory **(B,D,F)** populations, change with different coupling strengths for *g*, where *g* = 3 [**(A,B)**: both populations are chaotic], *g* = 1.9 [**(C,D)**: the inhibitory population is chaotic while the excitatory one is non-chaotic], and *g* = 1.5 [**(E,F)**: both populations are non-chaotic]. Here, the parameters are set as: *N*_*E*_ = 1,500, *N*_*I*_ = 1,000, *m*_*EE*_ = 1, *m*_*IE*_ = 0.75 *m*_*EI*_ = −0.25, *m*_*II*_ = −0.5, *σ* = 0.8, *η*_*E*_ = −1, and *η*_*I*_ = 0.5. The transfer function is selected as *ϕ*(*x*) = arctan*x*.

We use the numerical simulations to further illustrate the analytical results obtained in Proposition IV. 1. As shown in Figure 3A, when the transfer function is odd and the condition *η*_*E*_ *N*_*E*_ = *η*_*I*_ *N*_*I*_ is satisfied, the MLEs for the two populations are the same. This implies that the sensitivity of two populations depending on the initial values are the same, and also that the critical values for generating chaos in the two populations are the same. However, as shown in Figure 3B, when this condition is not satisfied, the MLEs for the two populations are different, which also indicates that the critical values for chaos emergence are likely different. Particularly, there are some coupling strengths for *g* (e.g., *g* = 1.9) such that one population behaves chaotically while the other one does not. This can be illustrated by the chaotic inhibitory population and the non-chaotic excitatory population, which are depicted in Figures 4C,D using the setting *g* = 1.9. Indeed, as shown in Figure 3C, the MLEs are the same although the transfer function is changed as a non-odd function. The same activation function is used in Figure 2C, where, however, the autocorrelation functions for the two populations are different. This reveals that, for calculating the MLEs, the condition *η*_*E*_ *N*_*E*_ = *η*_*I*_ *N*_*I*_ is probably robust against the selection of the transfer function. In addition, formula (14) is only a sufficient condition for chaos emergence in the two populations that behave in the same manner, and it cannot be expressed in a closed form and hard to be determined, since *c*_*K*0_ and ⟨*x*_*K*_⟩ satisfy Eqs 4, 9, 11. It is also worthwhile to mention that the MLEs, shown in Figure 3, for the two populations is not monotonically change with *g*, which is different from the previous results obtained in (Schuecker et al., 2018). Correspondingly, a more systematic view on how the dynamical behaviors of the two types of neurons change with *g* are depicted, respectively, in Figure 4.

**FIGURE 5:**
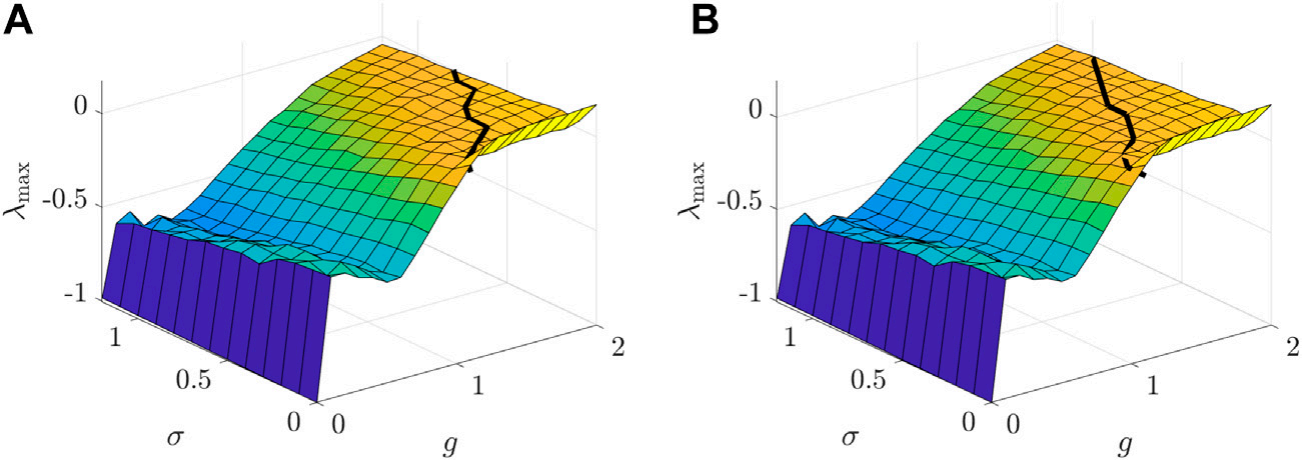
The MLEs change with *g*, the coupling strength coefficient, and *σ*, the intensity of white noise, respectively, for the population of excitatory neurons **(A)** and the population of inhibitory neurons **(B)**. The black lines mark *g*_*K*,*c*_, the critical points of two populations with given *σ*. The parameters are set as: *N*_*E*_ = 1,500, *N*_*I*_ = 1,000, *m*_*EE*_ = 1, *m*_*IE*_ = 0.75, *m*_*EI*_ = −0.25, *m*_*II*_ = −0.5, *η*_*E*_ = −1, and *η*_*I*_ = 0.5, and the transfer function is selected as *ϕ*(*x*) = arctanx.

**FIGURE 6:**
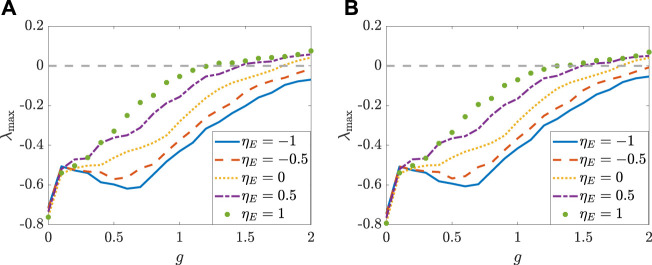
The MLEs change with *g*, the coupling strength coefficient, and *η*_*E*_, the correlation coefficient of excitatory population, respectively, for the population of the excitatory neurons **(A)** and the population of the inhibitory neurons **(B)**. The parameters are set as: *N*_*E*_ = 1,500, *N*_*I*_ = 1,000, *m*_*EE*_ = 1, *m*_*IE*_ = 0.75, *m*_*EI*_ = −0.25, *m*_*II*_ = −0.5, *σ* = 0.8 and *η*_*I*_ = 0, and the transfer function is selected as *ϕ*(*x*) = arctan*x*.

**FIGURE 7:**
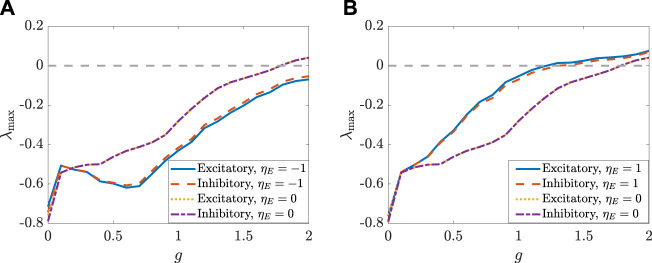
The MLEs change with *g*, the coupling strength coefficient, and *η*_*E*_, the correlation coefficient of excitatory population, respectively, for the two populations. The parameters are set as: *N*_*E*_ = 1,500, *N*_*I*_ = 1,000, *m*_*EE*_ = 1, *m*_*IE*_ = 0.75 *m*_*EI*_ = −0.25, *m*_*II*_ = −0.5, *σ* = 0.8 and *η*_*I*_ = 0, and the transfer function is selected as *ϕ*(*x*) = arctan*x*. **(A)** The comparison between *η*_*E*_ = −1 and *η*_*E*_ = 0. **(B)**The comparison between *η*_*E*_ = 1 and *η*_*E*_ = 0.

Figures 5, 6 depict how the intensity of white noise *σ* and the correlation coefficient *η*_*K*_ of the intra-connection influence the MLEs in addition to the coupling strength *g*. More precisely, as shown in Figure 5, the white noise with the intensity *σ* can suppress the onset of chaos for both populations of neurons. As for the noise-free case (i.e., *σ* = 0), the critical values *g*_*K*,*c*_ for chaos emergence are close to one; however, for the case with noise, *g*_*K*,*c*_ are larger than 1, which is consistent with the results obtained in (Schuecker et al., 2018). As shown in Figure 6, increasing correlation coefficient *η*_*K*_ of the intra-connection can promote chaos emergence. This is consistent with the findings for the single-population network where increasing the correlation coefficients of the connections yields a broader spectrum distribution of the adjacent matrix along the real axis and leads to chaos emergence (Girko, 1984; Sommers et al., 1988; Rajan and Abbott, 2006). As shown still in Figure 6, enlarging *η*_*K*_ renders the curve of the MLE more monotonously increasing. Additionally, changing *η*_*K*_ in one population can influence dynamics of both populations, but its impacts on the two MLEs are different. In particular, as shown in Figure 7, when *η*_*E*_ is above the value determined by the equation *η*_*E*_ *N*_*E*_ = *η*_*I*_ *N*_*I*_, it is more likely to enhance the chaos in the population of excitatory neurons.

**FIGURE 8:**
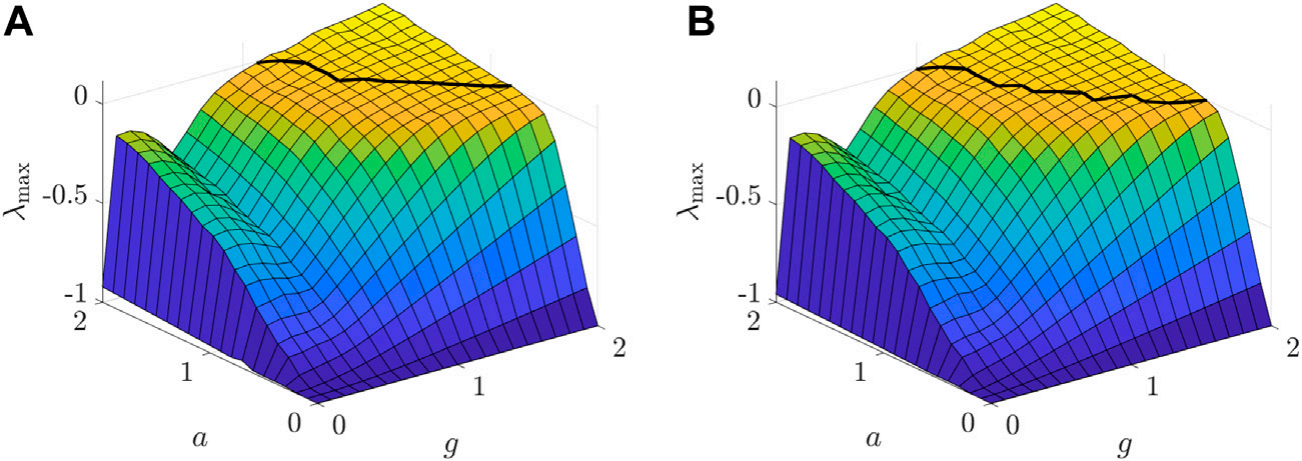
The MLEs change simultaneously with *g*, the coupling strength coefficient, and *a*, the slope in the transfer function *ϕ*_*a*_(*x*) = arctan(*ax*), respectively, for the population of excitatory neurons **(A)** and for the population of inhibitory neurons **(B)**. The black curves correspond to *g*_*K*,*c*_, the critical values for chaos occurrence in the two populations for different *a*. Here, the parameters are set as: *N*_*E*_ = 1,500, *N*_*I*_ = 1,000, *m*_*EE*_ = 1, *m*_*IE*_ = 0.75, *m*_*EI*_ = −0.25, *m*_*II*_ = −0.5, *η*_*E*_ = −1, and *η*_*I*_ = 1.

Also, we are to study how the setting in the activation function *ϕ* affects the phase transition of the studied neuronal networks. To this end, we introduce a parameter *a* into the transfer function as *ϕ*_*a*_(*x*) = arctan(*ax*). Clearly, the value of *a* determines the slope of the transfer function at the equilibrium. The larger the slope, the larger the absolute values of the eigenvalues in the vicinity of the equilibrium. As such, increasing the slope likely results in the instability of the neuronal network and promotes the chaos as well. This relation between the slope and the occurrence of chaos is demonstrated in Figure 8, where the numerical simulations on the MLEs and the critical value *g*_*K*,*c*_ for generating chaos are presented.

**FIGURE 9:**
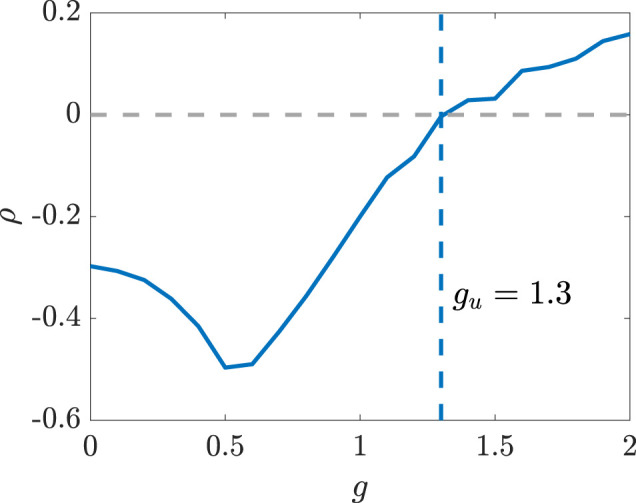
The quantity *ρ* changes with the parameters *g* and *η*_*E*_ for the two populations model. The parameters are set as: *N*_*E*_ = 1,500, *N*_*I*_ = 1,000, *m*_*EE*_ = 1, *m*_*IE*_ = 0.75, *m*_*EI*_ = −0.25, *m*_*II*_ = −0.5, *σ* = 0.8, *η*_*E*_ = −1 and *η*_*I*_ = 0.5, and the transfer function is selected as *ϕ*(*x*) = arctan*x*.

**FIGURE 10:**
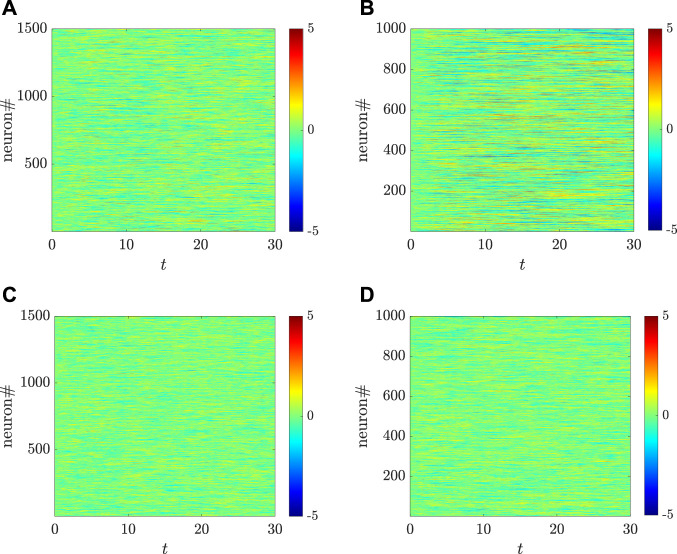
Different dynamical behaviors, respectively, for the excitatory **(A,C)** and the inhibitory **(B,D)** populations, change with different coupling strengths for *g*, where *g* = 1.5 (the first row: the linearly instability phase) and *g* = 0.8 (the second row: the linearly stable phase). Here, the parameters are set as: *N*_*E*_ = 1,500, *N*_*I*_ = 1,000, *m*_*EE*_ = 1, *m*_*IE*_ = 0.75 *m*_*EI*_ = −0.25, *m*_*II*_ = −0.5, *σ* = 0.8, *η*_*E*_ = −1, and *η*_*I*_ = 0.5. The transfer function is selected as *ϕ*(*x*) = arctan*x*.

Finally, the coexistence of linear instability and non-chaos, another interesting phenomenon, was reported for neural models having single population (Schuecker et al., 2018). Here, we validate that such a phenomenon is also present for the above two populations model, but it is crucially related to the selection of several parameters. More precisely, in order to analyze the linear stability of the model, we use its first-order approximation as follows:

$$\left\{\begin{array}{c} \frac{dz_{Ei}}{dt}=-z_{Ei}+\overset{N_{E}}{\underset{j=1}{\sum }}J_{Ei,Ej}\phi^{\prime }\left(x_{Ej}\right)z_{Ej}\left(t\right)+\overset{N_{I}}{\underset{j=1}{\sum }}J_{Ei,Ij}\phi^{\prime }\left(x_{Ij}\right)z_{Ij}\left(t\right),\\ \frac{dz_{Ii}}{dt}=-z_{Ii}+\overset{N_{E}}{\underset{j=1}{\sum }}J_{Ii,Ej}\phi^{\prime }\left(x_{Ej}\right)z_{Ej}\left(t\right)+\overset{N_{I}}{\underset{j=1}{\sum }}J_{Ii,Ij}\phi^{\prime }\left(x_{Ij}\right)z_{Ij}\left(t\right), \end{array}\right.$$ (15)

where *x*_*Ki*_ is the reference trajectory. The whole network is said to be linearly unstable as *ρ* is non-negative, where we denote by $\rho ≔max\left\{\right.(Re\left(\kappa \right)\;|\;\kappa$ the real part of the eigenvalue of the adjacent matrix specified in $\left(15\right)\left.\right\})$ . As numerically shown in Figure 9, the whole system is linearly stable until the coupling strength coefficient *g* goes beyond 1.3. However, as shown in Figure 3B, the populations of the excitatory and the inhibitory neurons display chaotic dynamics, respectively, at the critical points of *g* = *g*_*E*,*c*_ = 2 and *g* = *g*_*I*,*c*_ = 1.8, which are larger than 1.4. Hence, the phases of neither linear stability nor chaos exist still for the two populations model. Figure 10 shows the dynamical behaviors of the neither linearly stable nor chaotic phase and the linearly stable phase. Particularly, the regions of *g* for such phases in the respective populations are different: The population of the excitatory neurons having a larger value of the critical point corresponds to a broader range of *g*.

## 5 Discussion and Concluding Remarks

In this article, inspired by Sompolinsky’s framework on analyzing random neural networks, we have investigated a random neural network model containing both excitatory and inhibitory populations in large scales. We have taken into consideration not only the external perturbations of white noise, but also the randomness of inter- and intra-connections among the neurons. By applying the path integral formalism and the saddle-point approximation, we have reduced the model in large scales into a two-dimensional system with external Gaussian input. Based on the reduced model, we have depicted analytically and numerically how the parameters of populations and randomness influence the synchronization or/and chaotic dynamics of the two populations. Also, we have found different regions of the parameters with which the respective populations have dynamical behaviors of neither linear stability nor chaos.

As for the directions for the further study, we provide the following suggestions. First, although, along with the path integral, the methods used here are applicable to studying population dynamics in neuroscience (Zinn-Justin, 2002; Buice and Cowan, 2007; Chow and Buice, 2015), it is of tremendously tedious calculation to deal with systems having more than two populations. Thus, for multi-population, we suggest to seek additional mean-field methods (Ziegler, 1988; Olivier, 2008; Kadmon and Sompolinsky, 2015) for correspondingly deriving the closed-form equations for autocorrelation functions, obtaining the equivalent stochastic differential equations for the population-averaged Gaussian process, establishing the analytical conditions for synchronization emergence, and finding the phase diagram characterizing the regimes of multi-dynamics.

Second, in light of the spectrum theory of random matrices (Ginibre, 1965; Girko, 1984; Sommers et al., 1988; Tao et al., 2010; Nguyen and O’Rourke, 2014), for single population dynamics with or without external white noise, analytical criteria have been established for stable dynamics (Wainrib and Touboul, 2012) and for chaos emergence (Daniel et al., 2017) as well. In this article, we only use the numerical methods to roughly find the stability regions for the parameters, which suggests that, to obtain more accurate regions, we need to implement the analytical methods using the spectrum theory for random matrices even possessing some particular structures.

**FIGURE 11:**
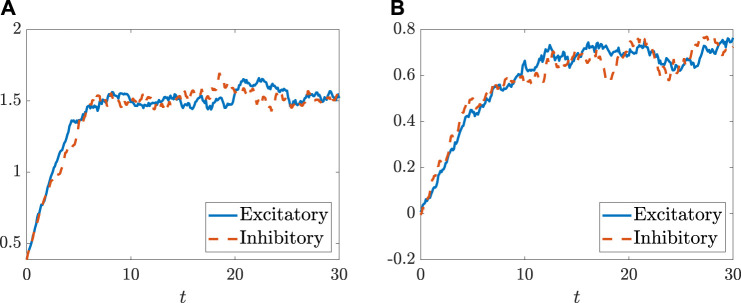
The autocorrelation functions ⟨*δx*_*E*_(*t*)*δx*_*E*_(*t* + *τ*)⟩ and ⟨*δx*_*I*_(*t*)*δx*_*I*_(*t* + *τ*)⟩ change with the evolution of the time *t*. The parameters are set as *η*_*E*_ *N*_*E*_ = *η*_*I*_ *N*_*I*_ = 500 with *N*_*E*_ = 1,500, *N*_*I*_ = 1,000, *m*_*EE*_ = 1, *m*_*IE*_ = 0.75, *m*_*EI*_ = −0.25, *m*_*II*_ = −0.5, *g* = 1.4, *σ* = 0.8, $\eta_{E}=\frac{1}{3}$ , and $\eta_{I}=\frac{1}{2}$ . The transfer function is set as *ϕ*(*x*) = arctan*x* and the time differences for the two numerical experiments are set as *τ* = 0 **(A)** and *τ* = 5 **(B)**, respectively.

Thirdly, we make an assumption that the process is stationary during our analytical investigation and characterize how neurons deviate from their stable states. During the numerical simulations, we treat this assumption through using the data of the processes after a sufficiently long period. As shown in Figure 11, such kind of treatments are reasonable as the processes go eventually stationary. However, the study of the transition to the stationary process or even the non-stationary processes, though rather difficult, could be the potential direction. Indeed, data-driven methods (Hou et al., 2022; Ying et al., 2022) could be the potential tools for studying these transition processes.

Finally, the coexistence of linearly instability and non-chaos is reported to be beneficial to enhance the information-processing capacity of the model of the single population neural network (Jaeger, 2002; Jaeger and Haas, 2004; Dambre et al., 2012; Schuecker et al., 2018). Here, we also find such a phenomenon in two populations model, and thus suggest to elucidate the relation between this phenomenon and the information-processing capacity in two populations model in the future study.

## Data Availability

The original contributions presented in the study are included in the article/Supplementary Material, further inquiries can be directed to the corresponding authors.
