# Supplementary material for: Complex Dynamics of Noise-Perturbed Excitatory-Inhibitory Neural Networks With Intra-Correlative and Inter-Independent Connections
Source: Front Physiol. 2022 Jun 24;13:915511. doi: 10.3389/fphys.2022.915511 (PMC9263264; doi:10.3389/fphys.2022.915511)
Supplement: Supplementary file 1 [file DataSheet1.pdf]

Supplementary Material
Appendices to "*Complex Dynamics of Noise-Perturbed Excitatory-Inhibitory Neural Networks with Intra-correlative and Inter-independent Connections*"

Xiaoxiao Peng[1,2,*] and Wei Lin[1,2,3,†]

[1] *Shanghai Center for Mathematical Sciences, School of Mathematical Sciences, and LMNS, Fudan University, Shanghai 200433, China*
[2] *Research Institute of Intelligent Complex Systems and Center for Computational Systems Biology, Fudan University, Shanghai 200433, China*
[3] *State Key Laboratory of Medical Neurobiology, and MOE Frontiers Center for Brain Science, Institutes of Brain Science, Fudan University, Shanghai 200032, China*

* xxpeng19@fudan.edu.cn
† wlin@fudan.edu.cn

CONTENTS

SA. Moment-generating functional with stationary Gaussian process 3

SB. Equivalent dynamic equation using mean field method and saddle-point approximation 5

SC. Differential equation for autocorrelation function 10

SD. Proofs of Propositions III.1 & III.2 10

SE. Equivalent dynamic equations for two dynamics 11

SF. Dynamic equation for deflection 13

SG. Proof of Proposition IV.1 14

SA. MOMENT-GENERATING FUNCTIONAL WITH STATIONARY GAUSSIAN PROCESS

Using the Martin-Siggia-Rose-de Dominicis-Janssen path integral formalism [9, 14, 31, 37], the following result has been obtained in [48]. However, for the completeness of this article, we provide the detailed arguments here.

We first consider the equation in one dimension that is written as

$$\mathrm{d}x(t) = f(t, x(t))\mathrm{d}t + N(t)\mathrm{d}t,$$

where $N(t)$ is the stationary Gaussian process with mean zero and satisfying $\langle N(t)N(t')\rangle = c(t,t')$. First, we perform the discretization for the above equation in the following manner:

$$x_i - x_{i-1} = f(t_{i-1}, x(t_{i-1}))\Delta t + N(t_{i-1})\Delta t,$$

where $\Delta t = t_i - t_{i-1}$ and t_0=0. Let $N_{i-1} := N(t_{i-1})$ and

$$y_i := x_{i-1} + f(t_{i-1}, x(t_{i-1}))\Delta t + N_{i-1}\Delta t.$$

Because of the property of the Dirac delta function, we derive

$$\begin{aligned} p(x_1, x_2, \cdots, x_M) &= \int \rho(N_0, N_1, \cdots, N_{M-1}) \prod_{i=1}^{M} \mathrm{d}N_{i-1}\delta(x_i - y_i(x_{i-1}, N_{i-1})) \\ &= \int \rho(N_0, N_1, \cdots, N_{M-1}) \prod_{i=1}^{M} \mathrm{d}N_{i-1}\delta(x_i - x_{i-1} - f(t_{i-1}, x(t_{i-1}))\Delta t - N_{i-1}\Delta t). \end{aligned}$$

Take the inverse Fourier transformation form of the Dirac delta function, we get

$$\delta(x) = \frac{1}{2\pi \mathrm{i}} \int_{-\mathrm{i}\infty}^{\mathrm{i}\infty} \mathrm{e}^{\tilde{x}x}\mathrm{d}\tilde{x}.$$

Hence,

$$\begin{aligned} p(x_1, x_2, \cdots, x_M) = \int \rho(N_0, N_1, \cdots, N_{M-1}) \prod_{i=1}^{M}\mathrm{d}N_{i-1} \int_{-\mathrm{i}\infty}^{\mathrm{i}\infty} \frac{\mathrm{d}\tilde{x}_i}{2\pi\mathrm{i}} \exp[\tilde{x}_i(x_i - x_{i-1} \\ -f(t_{i-1}, x(t_{i-1}))\Delta t - N_{i-1}\Delta t]. \end{aligned}$$

Define $D_{\boldsymbol{N}}(\tilde{\boldsymbol{x}}) := \langle \exp[-\Sigma_{i=1}^{M}\tilde{x}_i N_{i-1}\Delta t]\rangle_{\boldsymbol{N}}$, so that

$$D_{\boldsymbol{N}}(\tilde{\boldsymbol{x}}) = \frac{1}{(\sqrt{2\pi})^M\sqrt{|\boldsymbol{C}|}} \int_{R^M} \exp\left(-\frac{1}{2}\boldsymbol{N}^\top\boldsymbol{C}^{-1}\boldsymbol{N} - \tilde{\boldsymbol{x}}^\top\boldsymbol{N}\Delta t\right)\mathrm{d}\boldsymbol{N},$$

where $\boldsymbol{N} = (N_0, N_1, \cdots, N_{M-1})^\top$, $\tilde{x} = (\tilde{x}_E, \tilde{x}_I, \cdots, \tilde{x}_M)^\top$ and

$$\boldsymbol{C} = (c_{ij})_{i,j=1}^{M} = (c(t_i, t_j))_{i,j=0}^{M-1}.$$

Now, using the Cholesky decomposition for $\boldsymbol{C}$ yields $\boldsymbol{C} = \boldsymbol{B}\boldsymbol{B}^\top$, so that $\tilde{\boldsymbol{N}} = \boldsymbol{B}^{-1}\boldsymbol{N}$. This gives

$$D_{\boldsymbol{N}}(\tilde{\boldsymbol{x}}) = \frac{1}{(\sqrt{2\pi})^M\sqrt{|\boldsymbol{C}|}} \int_{R^M} |\boldsymbol{B}| \exp\left(-\frac{1}{2}\tilde{\boldsymbol{N}}^\top\tilde{\boldsymbol{N}} - \tilde{\boldsymbol{x}}^\top\boldsymbol{B}\tilde{\boldsymbol{N}}\Delta t\right)\mathrm{d}\tilde{\boldsymbol{N}}.$$

Notice that

$$\left|\boldsymbol{B}\boldsymbol{B}^\top\right| = |\boldsymbol{B}|^2 = |\boldsymbol{C}|.$$

Thus, the formula above becomes

$$\begin{aligned} &\frac{1}{(2\pi)^M} \int_{R^M} \mathrm{d}\tilde{\boldsymbol{N}} \exp\left[-\frac{1}{2}(\tilde{\boldsymbol{N}}^\top + \tilde{\boldsymbol{x}}^\top\boldsymbol{B}\Delta t)(\tilde{\boldsymbol{N}} + \boldsymbol{B}^\top\tilde{\boldsymbol{x}}\Delta t)\right) \exp\left[\frac{1}{2}\tilde{\boldsymbol{x}}^\top C\tilde{\boldsymbol{x}}(\Delta t)^2\right] \\ &= \exp\left[\frac{1}{2}\tilde{\boldsymbol{x}}^\top\boldsymbol{C}\tilde{\boldsymbol{x}}(\Delta t)^2\right] = \exp\left[\frac{1}{2}\sum_{i,j=1}^{M}\tilde{x}_i c(t_{i-1}, t_{j-1})\tilde{x}_j(\Delta t)^2)\right]. \end{aligned}$$

Now we introduce the source field $l=(l(t),t\in\mathbb{R})$ and consider $\boldsymbol{l}=(l_1,l_2,\cdots,l_M)$, where $l_i=l(t_i)$. Here, we study the characteristic function as follows:

$$Z[\boldsymbol{l}]=Z[l_1,l_2,\cdots,l_M]=\prod_{i=1}^{M}(\int_{-\infty}^{\infty}\exp(l_ix_i\Delta t)\mathrm{d}x_i)p(x_E,x_I,\cdots,x_M)$$
$$=\prod_{i=1}^{M}\left[\int_{-\infty}^{\infty}\exp(l_ix_i\Delta t)\mathrm{d}x_i\right)\int_{-\mathrm{i}\infty}^{\mathrm{i}\infty}\frac{\mathrm{d}\tilde{x}_i}{2\pi\mathrm{i}}\exp[\tilde{x}_i(x_i-x_{i-1}-f(t_{i-1},x(t_{i-1}))\Delta t)+\ln D_{\boldsymbol{N}}(\tilde{\boldsymbol{x}})].$$

Letting $\Delta t\to 0$ makes the formula continuous, which immediately yields:

$$\ln D_{\boldsymbol{N}}(\tilde{\boldsymbol{x}})\to\iint\frac{1}{2}\tilde{x}(t)C(t,t')\tilde{x}(t')\mathrm{d}t\mathrm{d}t'.$$

Consequently, we derive the moment-generating functional as follows:

$$Z[l(t)]=\int\mathfrak{D}x(t)\int\mathfrak{D}\tilde{x}(t)\ \exp(\tilde{x}^\top(\partial_t x-f(t,x(t)))+\frac{1}{2}\tilde{x}^\top C\tilde{x}+l^\top x),\tag{SA1}$$

where

$$\mathfrak{D}x(t):=\lim_{M\to\infty}\lim_{\Delta t\to 0}\prod_{i=1}^{M}\mathrm{d}x_i,$$

$$\mathfrak{D}\tilde{x}(t):=\lim_{M\to\infty}\lim_{\Delta t\to 0}\prod_{i=1}^{M}\frac{1}{2\pi\mathrm{i}}\mathrm{d}\tilde{x}_i,$$

$$\tilde{x}^\top C\tilde{x}:=\iint_{\mathbb{R}^2}\tilde{x}(t)C(t,t')\tilde{x}(t')\mathrm{d}t\mathrm{d}t',$$

$$\tilde{x}^\top(\partial_t x-f(t,x(t))):=\int_{\mathbb{R}}\tilde{x}(t)(\partial_t x(t)-f(t,x(t)))\mathrm{d}t,$$

$$l^\top x:=\int_{\mathbb{R}}l(t)x(t)\mathrm{d}t.$$

Particularly, as $N(t)$ is supposed to be the white noise satisfying $\langle N(t)N(t')\rangle=\sigma^2\delta(t-t')$, we have

$$Z[l(t)]=\int\mathfrak{D}x(t)\int\mathfrak{D}\tilde{x}(t)\ \exp\left[\tilde{x}^\top(\partial_t x-f(t,x(t)))+\frac{1}{2}\sigma^2\tilde{x}^\top\tilde{x}+l^\top x\right],$$

where

$$x^\top y=\int_{\mathbb{R}}x(t)y(t)\mathrm{d}t.$$

Moreover, for the system of higher-dimension which reads as

$$\mathrm{d}\boldsymbol{x}(t)=\boldsymbol{f}(t,\boldsymbol{x}(t))\mathrm{d}t+\sigma\boldsymbol{\xi}(t),$$

where $\boldsymbol{\xi}(t)$ is the standard white noise, the moment generating functional becomes

$$Z[\boldsymbol{l}(t)]=\int\mathfrak{D}\boldsymbol{x}(t)\int\mathfrak{D}\tilde{\boldsymbol{x}}(t)\ \exp\left[\tilde{\boldsymbol{x}}^\top(\partial_t\boldsymbol{x}-f(t,\boldsymbol{x}(t)))+\frac{1}{2}\sigma^2\tilde{\boldsymbol{x}}^\top\tilde{\boldsymbol{x}}+\boldsymbol{l}^\top\boldsymbol{x}\right],$$

which is akin to the functional obtained above for the one-dimensional system.

SB. EQUIVALENT DYNAMIC EQUATION USING MEAN FIELD METHOD AND SADDLE-POINT APPROXIMATION

Notice that

$$Z[\boldsymbol{l}](\boldsymbol{J}) = \int \mathfrak{D}\boldsymbol{x} \int \mathfrak{D}\tilde{\boldsymbol{x}} \ \exp\left\{S[\boldsymbol{x},\tilde{\boldsymbol{x}}] - \tilde{\boldsymbol{x}}^\top \boldsymbol{J}\phi(\boldsymbol{x}) + \boldsymbol{l}^\top \boldsymbol{x}\right\}. \tag{SB1}$$

We thus average the functional $Z[\boldsymbol{l}](\boldsymbol{J})$ with respect to $\boldsymbol{J}$ and obtain

$$\bar{Z}[\boldsymbol{l}] = \langle Z[\boldsymbol{l}](\boldsymbol{J})\rangle_J = \int Z[\boldsymbol{l}](\boldsymbol{J}) \ \mathcal{N}(\boldsymbol{M},\boldsymbol{A},\boldsymbol{J})\mathrm{d}\boldsymbol{J}.$$

Now, we calculate the coupling strength between the neurons in (SB1) which encompasses $J_{Ki,Lj}$. For the two neurons in the same population, we derive the term as

$$\begin{aligned}\frac{1}{2\pi\sqrt{|\boldsymbol{A}|}}\iint_{\mathbb{R}^2} \exp(-y_{Ki,Kj}J_{Ki,Kj} - y_{Kj,Ki}J_{Kj,Ki}) \exp\Bigg[-\frac{1}{2}\Bigg(J_{Ki,Kj} - \frac{m_{KK}}{N_K}, J_{Kj,Ki} \\ -\frac{m_{KK}}{N_K}\Bigg)\boldsymbol{A}_K{}^{-1}\begin{pmatrix} J_{Kj,Ki} - \frac{m_{KK}}{N_K} \\ J_{Ki,Kj} - \frac{m_{KK}}{N_K}\end{pmatrix}\Bigg]\mathrm{d}J_{Ki,Kj}\mathrm{d}J_{Kj,Ki}, \quad K\in\{E,I\},\end{aligned} \tag{SB2}$$

where

$$\boldsymbol{A}_K = g^2\begin{pmatrix} \frac{1}{N} & \frac{\eta_K}{N} \\ \frac{\eta_K}{N} & \frac{1}{N}\end{pmatrix}, \qquad y_{Ki,Lj} = \tilde{x}_{Ki}^\top \phi(x_{Kj}) = \int_{\mathbb{R}} \tilde{x}_{Ki}(t)\phi[x_{Kj}(t)]\mathrm{d}t.$$

Then, we apply the Cholesky decomposition to $\boldsymbol{A}_K$, obtaining

$$\boldsymbol{A}_K = \boldsymbol{B}_K\boldsymbol{B}_K{}^\top,\ \boldsymbol{B}_K = g\begin{pmatrix}\sqrt{\frac{1-\eta_K^2}{N}} & \frac{\eta_K}{\sqrt{N}} \\ 0 & \frac{1}{\sqrt{N}}\end{pmatrix}.$$

Letting $\boldsymbol{B}_K{}^{-1}\begin{pmatrix} J_{Ki,Kj} - \frac{m_{KK}}{N_K} \\ J_{Kj,Ki} - \frac{m_{KK}}{N_K}\end{pmatrix} = \begin{pmatrix}\tilde{J}_{Ki,Kj} \\ \tilde{J}_{Kj,Ki}\end{pmatrix}$ makes (SB2) become

$$\begin{aligned}&\frac{N}{2\pi g^2\sqrt{1-\eta_K^2}}\iint_{\mathbb{R}^2}\mathrm{d}\tilde{J}_{Ki,Kj}\mathrm{d}\tilde{J}_{Kj,Ki}\ \frac{g^2\sqrt{1-\eta_K^2}}{N}\exp\Bigg[-\frac{g}{\sqrt{N}}\sqrt{1-\eta_K^2}y_{Ki,Kj}\tilde{J}_{Ki,Kj} \\ &-\frac{g}{\sqrt{N}}\tilde{J}_{Kj,Ki}(y_{Ki,Kj}\eta_K + y_{Kj,Ki})\Bigg]\exp\Bigg[-\frac{1}{2}(\tilde{J}_{Ki,Kj}^2 + \tilde{J}_{Kj,Ki}^2)\Bigg]\exp\Bigg[-\frac{m_{KK}}{N_K}(y_{Ki,Kj}+y_{Kj,Ki})\Bigg] \\ &= \frac{1}{2\pi}\iint_{\mathbb{R}^2}\mathrm{d}\tilde{J}_{Ki,Kj}\mathrm{d}\tilde{J}_{Kj,Ki}\ \exp\Bigg[-\frac{1}{2}\Bigg(\tilde{J}_{Ki,Kj} + y_{Ki,Kj}g\sqrt{\frac{1-\eta_K^2}{N}}\Bigg)^2\Bigg] \\ &\exp\Bigg\{-\frac{1}{2}\Bigg[\tilde{J}_{Kj,Ki} + \frac{g}{\sqrt{N}}(y_{Ki,Kj}\eta_K + y_{Kj,Ki})\Bigg]^2\Bigg\}\exp\Bigg[\frac{g^2}{2N}(y_{Ki,Kj}^2 + y_{Kj,Ki}^2 \\ &+2y_{Ki,Kj}y_{Kj,Ki}\eta_K)\Bigg]\exp\Bigg[-\frac{m_{KK}}{N_K}(y_{Ki,Kj}+y_{Kj,Ki})\Bigg] \\ &= \exp\Bigg[\frac{g^2}{2N}(y_{Ki,Kj}^2 + y_{Kj,Ki}^2 + 2y_{Ki,Kj}y_{Kj,Ki}\eta_K)\Bigg]\exp\Bigg[-\frac{m_{KK}}{N_K}(y_{Ki,Kj}+y_{Kj,Ki})\Bigg].\end{aligned}$$

Additionally, for the two neurons in the different populations, we have that, as $K \neq L$,

$$\sqrt{\frac{N}{2\pi g^2}}\int_{\mathbb{R}} \mathrm{d}J_{Ki,Lj}\ \exp[-\tilde{x}_{Ki}^\top J_{Ki,Lj}\phi(x_{Lj})]\exp\left[-\frac{N(J_{Ki,Lj}-m_{KL}/N_L)^2}{2g^2}\right]$$
$$=\exp\left(-\frac{m_{KL}}{N_L}y_{Ki,Lj}+\frac{g^2}{2N}y_{Ki,Lj}^2\right).$$

Therefore,

$$\bar{Z}[\boldsymbol{l}]=\int\mathfrak{D}\boldsymbol{x}\int\mathfrak{D}\tilde{\boldsymbol{x}}\ \exp\Bigg\{S[\boldsymbol{x},\tilde{\boldsymbol{x}}]+\sum_{i\neq j\ \text{or}\ K\neq L}\left(-\frac{m_{KL}}{N_L}y_{Ki,Lj}+\frac{g^2}{2N}y_{Ki,Lj}^2\right)$$
$$+\sum_{K\in\{E,I\}}\sum_{i\neq j}\frac{g^2}{2N}y_{Ki,Kj}y_{Kj,Ki}\eta_K+\boldsymbol{l}^\top\boldsymbol{x}\Bigg\}.$$

To calculate the right side of the above quantity explicitly, we notice that the number of the elements corresponding to the diagonals are in the order of N^{-1}, compared to the remaining terms in the above quantity. Hence, we obtain

$$\exp\left\{\sum_{i\neq j\ \text{or}\ K\neq L}\left(-\frac{m_{KL}}{N_L}y_{Ki,Lj}+\frac{g^2}{2N}y_{Ki,Lj}^2\right)+\sum_{K\in\{E,I\}}\sum_{i\neq j}\frac{g^2}{2N}y_{Ki,Kj}y_{Kj,Ki}\eta_K\right\}$$
$$=\exp\left\{\sum_{i\neq j}\left(-k_{ij}y_{ij}+\frac{g^2}{2N}y_{ij}^2\right)+\sum_{K\in\{E,I\}}\sum_{i\neq j}\frac{g^2}{2N}y_{Ki,Kj}y_{Kj,Ki}\eta_K\right\}$$
$$\approx\exp\left\{\sum_{i,j}\left(-k_{ij}y_{ij}+\frac{g^2}{2N}y_{ij}^2\right)+\sum_{K\in\{E,I\}}\sum_{i,j}\frac{g^2}{2N}y_{Ki,Kj}y_{Kj,Ki}\eta_K\right\}$$
$$=\exp\Bigg\{\frac{g^2}{2N}\Bigg[\sum_{i,j=1}^{N}\iint_{\mathbb{R}^2}\tilde{x}_i(t)\phi(x_j(t))\tilde{x}_i(t')\phi(x_j(t'))\mathrm{d}t\mathrm{d}t'$$
$$+\sum_{K\in\{E,I\}}\sum_{i,j}\iint_{\mathbb{R}^2}\eta_K\tilde{x}_{Ki}(t)\phi(x_{Kj}(t))\tilde{x}_{Kj}(t')\phi(x_{Ki}(t'))\mathrm{d}t\mathrm{d}t'-\sum_{i,j=1}^{N}\int_{\mathbb{R}}k_{ij}\tilde{x}_i(t)\phi(x_j(t))\mathrm{d}t\Bigg]\Bigg\},$$

where the subscripts K and L, denoting the two populations, are omitted for simplicity and

$$x_i(t)=\begin{cases}x_{E,i}(t), & 1\leqslant i\leqslant N_E,\\ x_{I,i-N_E}(t), & N_E+1\leqslant i\leqslant N,\end{cases}$$

$$k_{ij}=\begin{cases}m_{EE}/N_E, & i,j\leq N_E,\\ m_{IE}/N_E, & j\leq N_E, i>N_E,\\ m_{EI}/N_I, & i\leq N_E, j>N_E,\\ m_{II}/N_I, & i,j>N_E,\end{cases}\qquad y_{ij}=\begin{cases}y_{Ei,Ej}, & i,j\leq N_E,\\ y_{I(i-N_E),Ej}, & j\leq N_E, i>N_E,\\ y_{Ei,I(j-N_E)}, & i\leq N_E, j>N_E,\\ y_{E(i-N_E),I(j-N_E)}, & i,j>N_E.\end{cases}$$

In what follows, we use the notations:

$$Q(t,t')=\frac{g^2}{N}\sum_{j=1}^{N}\phi(x_j(t))\phi(x_j(t')),$$
$$R_E(t)=\frac{1}{N_E}\sum_{i=1}^{N_E}\phi(x_{Ei}(t)),\ \ R_I(t)=\frac{1}{N_I}\sum_{i=1}^{N_I}\phi(x_{Ii}(t)),\tag{SB3}$$
$$T_E(t,t')=\frac{g^2\eta_E}{N}\sum_{j=1}^{N_E}\phi(x_{1,j}(t))\tilde{x}_{1,j}(t'),\quad T_I(t,t')=\frac{g^2\eta_I}{N}\sum_{j=1}^{N_E}\phi(x_{2,j}(t))\tilde{x}_{2,j}(t').$$

Then, we have

$$\bar{Z}[\boldsymbol{l}] = \int \mathfrak{D}\boldsymbol{x} \int \mathfrak{D}\tilde{\boldsymbol{x}} \; \exp\Big\{ S[\boldsymbol{x},\tilde{\boldsymbol{x}}] + \boldsymbol{l}^\top \boldsymbol{x} + \frac{1}{2}\tilde{\boldsymbol{x}}^\top Q \tilde{\boldsymbol{x}} - m_{EE}\tilde{\boldsymbol{x}}_E^\top R_E \mathbf{1} - m_{IE}\tilde{\boldsymbol{x}}_I^\top R_E \mathbf{1} - m_{EI}\tilde{\boldsymbol{x}}_E^\top R_I \mathbf{1} - m_{II}\tilde{\boldsymbol{x}}_I^\top R_I \mathbf{1} + \frac{1}{2}\tilde{\boldsymbol{x}}_E^T T_E \boldsymbol{\phi}(x_E) + \frac{1}{2}\tilde{\boldsymbol{x}}_I^T T_I \boldsymbol{\phi}(x_I) \Big\}, \tag{SB4}$$

where we define $\tilde{\boldsymbol{x}}^\top Q \tilde{\boldsymbol{x}} = \sum_{i=1}^N \iint \tilde{x}_i(t) Q(t,t') \tilde{x}_i(t') \mathrm{d}t \mathrm{d}t'$ and define the remaining terms in (SB4) in an analogous manner.

Next, we are to calculate Q, R_K, and T_K, more explicitly. To this end, from the property of the Dirac delta function, the quantity in (SB4) is rewritten as

$$\begin{aligned}\bar{Z}[\boldsymbol{l}] = \int \mathfrak{D}\boldsymbol{x} \int \mathfrak{D}\tilde{\boldsymbol{x}} \int \mathfrak{D}Q \int \mathfrak{D}R_E \int \mathfrak{D}R_I \int \mathfrak{D}T_E \int \mathfrak{D}T_I \; \exp\Bigg\{ S[\boldsymbol{x},\tilde{\boldsymbol{x}}] + \boldsymbol{l}^\top \boldsymbol{x} + \frac{1}{2}\tilde{\boldsymbol{x}}^\top Q \tilde{\boldsymbol{x}} \\ - m_{EE}\tilde{\boldsymbol{x}}_E^\top R_E \mathbf{1} - m_{IE}\tilde{\boldsymbol{x}}_I^\top R_E \mathbf{1} - m_{EI}\tilde{\boldsymbol{x}}_E^\top R_I \mathbf{1} - m_{II}\tilde{\boldsymbol{x}}_I^\top R_I \mathbf{1} + \frac{1}{2}\tilde{\boldsymbol{x}}_E^T T_E \boldsymbol{\phi}(x_E) + \frac{1}{2}\tilde{\boldsymbol{x}}_I^T T_I \boldsymbol{\phi}(x_I) \Bigg\} \\ \frac{N}{g^2}\delta\Bigg\{ - \frac{N}{g^2} Q + \sum_{i=1}^N \phi(x_i(t))\phi(x_i(t')) \Bigg] N_E \delta\Bigg[- N R_E + \sum_{i=1}^{N_E} \phi(x_{Ei}(t)) \Bigg] \\ N_I \delta\Bigg[- N R_I + \sum_{i=1}^{N_I} \phi(x_{Ii}(t)) \Bigg] \frac{N}{g^2\eta_E}\delta\Bigg[- \frac{N}{g^2\eta_E} T_E + \sum_{i=1}^{N_E} \phi(x_{Ei}(t))\tilde{x}_{Ei}(t') \Bigg] \\ \frac{N}{g^2\eta_I}\delta\Bigg[- \frac{N}{g^2\eta_I} T_I + \sum_{i=1}^{N_I} \phi(x_{Ei}(t))\tilde{x}_{Ei}(t') \Bigg].\end{aligned}$$

Then, we write the Dirac delta function in a form of the Fourier transformation as

$$\delta\Bigg[- \frac{N}{g^2} Q(t,t') + \sum_{i=1}^N \phi(x_i(t))\phi(x_i(t')) \Bigg] = \int \mathfrak{D}\tilde{Q} \; \exp\Bigg[- \frac{N}{g^2} Q^\top \tilde{Q} + \sum_{i=1}^N \phi(x_i)^\top \tilde{Q} \phi(x_i) \Bigg],$$

where $\tilde{Q}$ is an imaginary field and $Q^\top \tilde{Q} = \iint \mathrm{d}t\mathrm{d}t' Q(t,t')\tilde{Q}(t,t')$. Analogously,

$$\begin{aligned}\delta\Bigg[- N_E R_E(t) + \sum_{i=1}^{N_E} \phi(x_{Ei}(t)) \Bigg] &= \int \mathfrak{D}\tilde{R}_E \; \exp\Bigg[- N_E R_E^\top \tilde{R}_E + \sum_{i=1}^{N_E} \phi(x_{Ei})^\top \tilde{R}_E \Bigg], \\ \delta\Bigg[- N_I R_I(t) + \sum_{i=1}^{N_I} \phi(x_{Ii}(t)) \Bigg] &= \int \mathfrak{D}\tilde{R}_I \; \exp\Bigg[-N_I R_I^\top \tilde{R}_I + \sum_{i=1}^{N_I} \phi(x_{Ii})^\top \tilde{R}_I \Bigg], \\ \delta\Bigg[- \frac{N}{g^2\eta_E} T_E(t,t') + \sum_{i=1}^{N_E} \phi(x_{Ei}(t))\tilde{x}_{Ei}(t') \Bigg] &= \int \mathfrak{D}\tilde{T}_E \exp\Bigg[- \frac{N}{g^2\eta_E} T_E^\top \tilde{T}_E + \sum_{i=1}^{N_E} \phi(x_{Ei})^\top \tilde{T}_E \tilde{x}_{Ei} \Bigg], \\ \delta\Bigg[- \frac{N}{g^2\eta_I} T_I(t,t') + \sum_{i=1}^{N_I} \phi(x_{Ii}(t))\tilde{x}_{Ii}(t') \Bigg] &= \int \mathfrak{D}\tilde{T}_I \; \exp\Bigg[- \frac{N}{g^2\eta_I} T_I^\top \tilde{T}_I + \sum_{i=1}^{N_I} \phi(x_{Ii})^\top \tilde{T}_I \tilde{x}_{Ii} \Bigg],\end{aligned}$$

Then, we obtain

$$\bar{Z} \propto \int \mathfrak{D}Q\mathfrak{D}\tilde{Q} \prod_{K\in\{E,I\}} \Big(\mathfrak{D}R_K \mathfrak{D}\tilde{R}_K \mathfrak{D}T_K \mathfrak{D}\tilde{T}_K \Big) \; \exp\Bigg[- \frac{N}{g^2} Q^\top \tilde{Q} + \sum_{K\in\{E,I\}} \Bigg(- N_K R_K^\top \tilde{R}_K - \frac{N}{g^2\eta_K} T_K^\top \tilde{T}_K + N_K \ln \omega_K \Bigg) + l_Q^\top Q + l_{\tilde{Q}}^\top \tilde{Q} + \sum_{K\in\{E,I\}} \Bigg(l_{R_K}^\top R_K + l_{\tilde{R}_K}^\top \tilde{R}_K + l_{T_K}^\top T_K + l_{\tilde{T}_K}^\top \tilde{T}_K \Bigg) \Bigg], \tag{SB5}$$

where

$$\omega_E = \int \mathfrak{D}x_E \int \mathfrak{D}\tilde{x}_E \ \exp\Bigg\{ - m_{EE}\tilde{x}_E^\top R_E - m_{EI}\tilde{x}_E^\top R_I + \phi(x_E)^\top \tilde{R}_E + S[x_E,\tilde{x}_E]$$
$$+ \frac{1}{2}\tilde{x}_E^\top Q\tilde{x}_E + \phi(x_E)^\top \tilde{Q}\phi(x_E) + \frac{1}{2}\tilde{x}_E^\top T_E\phi(x_E) + \phi(x_E)^\top \tilde{T}_E\tilde{x}_E\Bigg\},$$
$$\omega_I = \int \mathfrak{D}x_I \int \mathfrak{D}\tilde{x}_I \ \exp\Bigg\{ - m_{IE}\tilde{x}_I^\top R_E - m_{II}\tilde{x}_I^\top R_I + \phi(x_I)^\top \tilde{R}_I + S[x_I,\tilde{x}_I]$$
$$+ \frac{1}{2}\tilde{x}_I^\top Q\tilde{x}_I + \phi(x_I)^\top \tilde{Q}\phi(x_I) + \frac{1}{2}\tilde{x}_I^\top T_I\phi(x_I) + \phi(x_I)^\top \tilde{T}_I\tilde{x}_I\Bigg\}.$$

Since there is no physical meaning for the source field, we omit it in the following calculations.

When N is sufficiently large, we apply the saddle point approximation to $\bar{Z}$, which yields:

$$\bar{Z}^* \propto \exp\Bigg(- \frac{N}{g^2}Q^{*\top}\tilde{Q}^* - N_E R_E^{*\top}\tilde{R}_E^* - N_I R_I^{*\top}\tilde{R}_I^* - \frac{N}{g^2\eta_E}T_E^{*\top}\tilde{T}_E^* - \frac{N}{g^2\eta_I}T_I^{*\top}\tilde{T}_I^*$$
$$+N_E \ln\omega_E[Q^*,\tilde{Q}^*,R_K^*,\tilde{R}_K^*,T_K^*,\tilde{T}_K^*] + N_I\ln\omega_I[Q^*,\tilde{Q}^*,R_K^*,\tilde{R}_K^*,T_K^*,\tilde{T}_K^*]\Bigg). \tag{SB6}$$

Here, all the parameters with the star superscripts in (SB6) satisfy

$$\frac{\delta S}{\delta[Q,\tilde{Q},R_E,\tilde{R}_E,R_I,\tilde{R}_I,T_E,\tilde{T}_E,T_I,\tilde{T}_I]} = 0,$$

where δ represents the variation with respect to the corresponding quantity and

$$S = -\frac{N}{g^2}Q^\top\tilde{Q} - N_E R_E^\top\tilde{R}_E - N_I R_I^\top\tilde{R}_I - \frac{N}{g^2\eta_E}T_E^\top\tilde{T}_E - \frac{N}{g^2\eta_I}T_I^\top\tilde{T}_I + N_E\ln\omega_E + N_I\ln\omega_I.$$

For Q^*, we get

$$0 = -\frac{N}{g^2}Q^* + \frac{N_E}{\omega_E}\frac{\delta\omega_E}{\delta\tilde{Q}} + \frac{N_I}{\omega_I}\frac{\delta\omega_I}{\delta\tilde{Q}}.$$

Denote, respectively, by

$$\omega_1 = \int \mathfrak{D}x_E \int \mathfrak{D}\tilde{x}_E P,$$

and

$$\langle f\rangle_{\omega,E} = \frac{\int\mathfrak{D}x_E\int\mathfrak{D}\tilde{x}_E Pf}{\int\mathfrak{D}x_E\int\mathfrak{D}\tilde{x}_E P},$$

where $\langle f\rangle_{\omega,E}$ (resp., $\langle f\rangle_{\omega,I}$) stands for the average value of f in a sense of the excitatory (resp., inhibitory) population in a large scale. Then,

$$Q^* = g^2\frac{N_E\langle\tilde{x}_E(t)\tilde{x}_E(t')\rangle_{\omega,E} + N_I\langle\tilde{x}_I(t)\tilde{x}_I(t')\rangle_{\omega,I}}{N}.$$

As $\tilde{x}$ is the imaginary field derived from the Fourier transformation of the Dirac delta function, we stipulate that its expectation is zero. This stipulation is physically reasonable, so that $\tilde{Q}^* = 0$. Similarly, we get

$$R_E^* = \langle\phi(x_E(t))\rangle_{\omega,E}, \qquad R_I^* = \langle\phi(x_I(t))\rangle_{\omega,I},$$
$$T_E^* = \frac{g^2\eta_E N_E}{N}\langle\phi(x_E(t))\tilde{x}_E(t')\rangle_{\omega,E}, \qquad \tilde{T}_E^* = \frac{g^2\eta_E N_E}{2N}\langle\tilde{x}_E(t)\phi(x_E(t'))\rangle_{\omega,I},$$
$$T_I^* = \frac{g^2\eta_I N_I}{N}\langle\phi(x_I(t))\tilde{x}_I(t')\rangle_{\omega,E}, \qquad \tilde{T}_I^* = \frac{g^2\eta_I N_I}{2N}\langle\tilde{x}_I(t)\phi(x_I(t'))\rangle_{\omega,I},$$
$$\tilde{R}_E^* = \tilde{R}_I^* = 0.$$

In the following the subscript of the expectation $\langle\cdot\rangle$ is omitted. Let

$$O_K(t,t') = \langle \phi(x_K(t))\phi(x_K(t'))\rangle,\ \ F_K(t,t') = \langle \tilde{x}_K(t)\phi(x_K(t'))\rangle,\ \ K\in\{E,I\}.$$

Then,

$$\begin{aligned}\bar{Z}^* \propto & \left\{\int \mathfrak{D}x_E \int \mathfrak{D}\tilde{x}_E\ \ \exp\left\{\frac{g^2 N_E\eta_E}{2N}[-F_E(t,t')^\top F_E(t',t) + 2\phi(x_E(t))^\top F_E(t,t')\tilde{x}_E(t')]\right.\right. \\ & + S[x_E,\tilde{x}_E] + \frac{g^2}{2N}[N_E\tilde{x}_E(t)^\top O_E(t,t')\tilde{x}_E(t') + N_I\tilde{x}_E(t)^\top O_I(t,t')\tilde{x}_E(t')] - m_{EE}\tilde{x}_E^\top\langle\phi(x_E(t))\rangle \\ & \left.\left. - m_{EI}\tilde{x}_E^\top\langle\phi(x_I(t))\rangle\right\}\right\}^{N_E}\left\{\int \mathfrak{D}x_I \int \mathfrak{D}\tilde{x}_I \exp\left\{\frac{g^2 N_I\eta_I}{2N}[-F_I(t,t')^\top F_I(t',t)\right.\right. \\ & + 2\phi(x_I(t))^\top F_I(t,t')\tilde{x}_I(t')] + S[x_I,\tilde{x}_I] + \frac{g^2}{2N}[N_E\tilde{x}_I(t)^\top O_E(t,t')\tilde{x}_I(t') \\ & \left.\left. + N_I\tilde{x}_I(t)^\top O_I(t,t')\tilde{x}_I(t')] - m_{IE}\tilde{x}_I^\top\langle\phi(x_E(t))\rangle - m_{II}\tilde{x}_I^\top\langle\phi(x_I(t))\rangle\right\}\right\}^{N_I}.\end{aligned}$$

When N is sufficiently large, we have

$$\begin{aligned}& 2\phi(x(t))^\top F(t,t')\tilde{x}(t') - F(t,t')^\top F(t',t) \\ = & \iint [2\phi(x(t))\langle\tilde{x}(t)\phi(x(t'))\rangle\tilde{x}(t') - \langle\tilde{x}(t')\phi(x(t))\rangle\langle\tilde{x}(t)\phi(x(t'))\rangle]\mathrm{d}t\mathrm{d}t' \\ \approx & \iint [2\phi(x(t))\tilde{x}(t)\phi(x(t'))\tilde{x}(t') - \tilde{x}(t')\phi(x(t))\tilde{x}(t)\phi(x(t'))]\mathrm{d}t\mathrm{d}t' \\ = & \iint \phi(x(t))\tilde{x}(t)\phi(x(t'))\tilde{x}(t')\mathrm{d}t\mathrm{d}t' \\ \approx & \iint \tilde{x}(t)\langle\phi(x(t))\phi(x(t'))\rangle\tilde{x}(t')\mathrm{d}t\mathrm{d}t',\end{aligned}$$

which implies that

$$\begin{aligned}\bar{Z}^* \propto & \left\{\int \mathfrak{D}x_E \int \mathfrak{D}\tilde{x}_E\ \ \exp\left\{\frac{g^2 N_E(\eta_E+1)}{2N}\tilde{x}_E(t)\langle\phi(x_E(t))\phi(x_E(t'))\rangle\tilde{x}_E(t') + S[x_E,\tilde{x}_E]\right.\right. \\ & \left.\left. + \frac{g^2 N_I}{2N}\tilde{x}_E(t)^\top\langle\phi(x_I(t))\phi(x_I(t'))\rangle\tilde{x}_E(t') - m_{EE}\tilde{x}_E^\top\langle\phi(x_E(t))\rangle - m_{EI}\tilde{x}_E^\top\langle\phi(x_I(t))\rangle\right\}\right\}^{N_E} \\ & \left\{\int \mathfrak{D}x_I \int \mathfrak{D}\tilde{x}_I \exp\left\{\frac{g^2 N_I(\eta_I+1)}{2N}\tilde{x}_I(t)\langle\phi(x_I(t))\phi(x_I(t'))\rangle\tilde{x}_I(t') + S[x_I,\tilde{x}_I]\right.\right. \\ & \left.\left. + \frac{g^2 N_E}{2N}\tilde{x}_I(t)^\top\langle\phi(x_E(t))\phi(x_E(t'))\rangle\tilde{x}_I(t') - m_{IE}\tilde{x}_I^\top\langle\phi(x_E(t))\rangle - m_{II}\tilde{x}_I^\top\langle\phi(x_I(t))\rangle\right\}\right\}^{N_I}.\end{aligned}$$

Thus, together with the results obtained in (SA1) of Appendix SA, the formula above becomes the moment-generating functional for N_E identical excitatory neurons and N_I identical inhibitory neurons with the external Gaussian process. Correspondingly, the dynamical equations become:

$$\begin{cases}\dfrac{\mathrm{d}x_E}{dt} &= -x + \gamma_E(t) + \sigma\xi_E(t) + m_{EE}\langle\phi(x_E(t))\rangle + m_{EI}\langle\phi(x_I(t))\rangle, \\ \dfrac{\mathrm{d}x_I}{dt} &= -x + \gamma_I(t) + \sigma\xi_I(t) + m_{IE}\langle\phi(x_E(t))\rangle + m_{II}\langle\phi(x_I(t))\rangle.\end{cases}$$

where $\xi_K(t)$ with $K\in\{E,I\}$ are the mutually independent white noises and $\gamma_K(t)$ with $K\in\{E,I\}$ are the Gaussian processes with mean zeros satisfying

$$\begin{aligned}\langle\gamma_E(t)\gamma_E(t')\rangle &= \frac{g^2}{N}[N_E(1+\eta_E)\langle\phi(x_E(t))\phi(x_E(t'))\rangle + N_I\langle\phi(x_I(t))\phi(x_I(t'))\rangle], \\ \langle\gamma_I(t)\gamma_I(t')\rangle &= \frac{g^2}{N}[N_E\langle\phi(x_E(t))\phi(x_E(t'))\rangle + N_I(1+\eta_I)\langle\phi(x_I(t))\phi(x_I(t'))\rangle].\end{aligned}$$

This therefore completes the derivation of the equation that we anticipate above.

SC. DIFFERENTIAL EQUATION FOR AUTOCORRELATION FUNCTION

Substitution of (4) into (3) gives

$$\frac{\mathrm{d}\delta x_K}{\mathrm{d}t} = -\delta x_K + \gamma_K(t) + \sigma\xi_K(t), \quad K \in \{E, I\}.$$

Then, we have

$$(\partial_t + 1)(\partial_{t'} + 1)\langle \delta x_K(t)\delta x_K(t')\rangle = \langle \gamma_K(t)\gamma_K(t')\rangle + \sigma^2\langle \xi_K(t)\xi_K(t')\rangle. \tag{SC1}$$

Through setting $t' = t + \tau$ in (SC1) and using an assumption that the neurons' states in the two populations are the stationary Gaussian processes, we obtain

$$(-\partial_\tau^2 + 1)\langle \delta x_K(t)\delta x_K(t+\tau)\rangle = \langle \gamma_K(t)\gamma_K(t+\tau)\rangle + \sigma^2\langle \xi_K(t)\xi_K(t+\tau)\rangle,$$

which yields:

$$\begin{aligned}\frac{\mathrm{d}^2 C_E}{\mathrm{d}\tau^2} &= C_E - \frac{g^2}{N}[N_E(\eta_E+1)\langle\phi(x_E(t))\phi(x_E(t+\tau))\rangle + N_I\langle\phi(x_I(t))\phi(x_I(t+\tau))\rangle] - \sigma^2\delta(\tau),\\ \frac{\mathrm{d}^2 C_I}{\mathrm{d}\tau^2} &= C_I - \frac{g^2}{N}[N_E\langle\phi(x_E(t))\phi(x_E(t+\tau))\rangle + N_I(\eta_I+1)\langle\phi(x_I(t))\phi(x_I(t+\tau))\rangle] - \sigma^2\delta(\tau).\end{aligned}$$

SD. PROOFS OF PROPOSITIONS III.1 & III.2

Proof of Proposition III.1: Using $f_{\phi(\cdot+\langle x_K\rangle)}(C_K, c_{K0})$ renders (5) as

$$\begin{cases}\dfrac{\mathrm{d}^2 C_E}{\mathrm{d}\tau^2} = C_E - \frac{g^2 N_E(1+\eta_E)}{N} f_{\phi(\cdot+\langle x_E\rangle)}(C_E, c_{E0}) - \frac{g^2 N_I}{N} f_{\phi(\cdot+\langle x_I\rangle)}(C_I, c_{I0}) - \sigma^2\delta_E(\tau),\\ \dfrac{\mathrm{d}^2 C_I}{\mathrm{d}\tau^2} = C_I - \frac{g^2 N_E}{N} f_{\phi(\cdot+\langle x_E\rangle)}(C_E, c_{E0}) - \frac{g^2 N_I(1+\eta_I)}{N} f_{\phi(\cdot+\langle x_I\rangle)}(C_I, c_{I0}) - \sigma^2\delta_I(\tau),\\ C_E(0) = c_{E0},\ C_I(0) = c_{I0}.\end{cases} \tag{SD1}$$

Define $W_{E,I}$ in the manner as those defined in (10). Thus, we have

$$\begin{cases}\dfrac{\mathrm{d}^2 C_E}{\mathrm{d}\tau^2} = W_E(C_E, C_I; c_{E0}, c_{I0}) - \sigma^2\delta_E(\tau),\\ \dfrac{\mathrm{d}^2 C_I}{\mathrm{d}\tau^2} = W_I(C_E, C_I; c_{E0}, c_{I0}) - \sigma^2\delta_I(\tau).\end{cases}$$

Consider the step function as

$$\varepsilon(t) = \begin{cases}\frac{1}{2}, & t \geqslant 0,\\ -\frac{1}{2}, & t < 0,\end{cases}$$

whose derivative is the Dirac delta function, a generalized function. As $C_{E,I}$ is even from its definition, it is reasonable to assume that

$${C_E}'(0+) = {C_I}'(0+) = -\frac{1}{2}\sigma^2.$$

Define

$$\rho_K(x) = \int_0^x \phi(y + \langle x_K\rangle)\mathrm{d}y.$$

By virtue of Price's Theorem [45], we have

$$\begin{cases}\dfrac{\partial}{\partial C_E} f_{\rho_E}(C_E, c_{E0}) = f_{\phi(\cdot+\langle x_E\rangle)}(C_E, c_{E0}),\\ \dfrac{\partial}{\partial C_I} f_{\rho_I}(C_I, c_{I0}) = f_{\phi(\cdot+\langle x_I\rangle)}(C_I, c_{I0}).\end{cases}$$

Hence, with

$$\begin{cases} V_E(C_E, C_I; c_{E0}, c_{I0}) & = -\frac{1}{2}C_E^2 + \frac{g^2 N_E(1+\eta_E)}{N} f_{\rho_E}(C_E, c_{E0}) + \frac{g^2 N_I}{N} f_{\rho_I}(C_I, c_{I0}), \\ V_I(C_E, C_I; c_{E0}, c_{I0}) & = -\frac{1}{2}C_I^2 + \frac{g^2 N_E}{N} f_{\rho_E}(C_E, c_{E0}) + \frac{g^2 N_I(1+\eta_I)}{N} f_{\rho_I}(C_I, c_{I0}), \end{cases}$$

we obtain

$$\begin{cases} \frac{1}{2}{C_E'}^2 + V_E(C_E, C_I) & = \text{const}, \\ \frac{1}{2}{C_I'}^2 + V_I(C_E, C_I) & = \text{const}. \end{cases}$$

With an additional assumption that the autocorrelation functions tend towards a constant as τ goes to infinity, we have

$$\begin{cases} \frac{\sigma^4}{8} + V_E(c_{E0}, c_{I0}) = V_E(c_{E\infty}, c_{I\infty}), \\ \frac{\sigma^4}{8} + V_I(c_{E0}, c_{I0}) = V_I(c_{E\infty}, c_{I\infty}) \end{cases}$$

and

$$W_E(c_{E\infty}, c_{I\infty}; c_{E0}, c_{I0}) = W_I(c_{E\infty}, c_{I\infty}; c_{E0}, c_{I0}) = 0,$$

which completes the analytical validation of this proposition.

Proof of Proposition III.2: (1) When the transfer function is odd, together with $W_{E,I}$ defined in (10), the definition of $f_{\phi(\cdot+\langle x_{E,I}\rangle)}(C_{E,I}(\tau), c_{E,I0})$ (6) and $\langle x_E \rangle = \langle x_I \rangle = 0$, we immediately have

$$W_E(0, 0; c_{E0}, c_{I0}) = W_I(0, 0; c_{E0}, c_{I0}) = 0.$$

Thus, we claim that $c_{E\infty} = c_{I\infty} = 0$. If, additionally, $\sigma = 0$, then it follows from the formula (9) that $c_{E0} = c_{I0} = 0$.

(2) Actually, when one of the three conditions assumed in the proposition is satisfied, the two equations describing the dynamics of C_E and C_I in (SD1) are identical. Moreover, due to (9) and (11), we have $c_{E0} = c_{I0}$. Therefore, we conclude that the values of C_E and C_I are identical for all τ.

SE. EQUIVALENT DYNAMIC EQUATIONS FOR TWO DYNAMICS

For the moment-generating functional

$$Z[\boldsymbol{l}^1, \boldsymbol{l}^2](\boldsymbol{J}) = \prod_{\alpha=1}^{2} \left\{ \int \mathfrak{D}\boldsymbol{x}^\alpha \int \mathfrak{D}\tilde{\boldsymbol{x}}^\alpha \exp\{S[\boldsymbol{x}^\alpha, \tilde{\boldsymbol{x}}^\alpha] - \tilde{\boldsymbol{x}}^{\alpha\top}\boldsymbol{J}\phi(\boldsymbol{x}^\alpha) + \boldsymbol{l}^{\alpha\top}\boldsymbol{x}^\alpha\} \right\} \exp(\sigma^2 \tilde{\boldsymbol{x}}^{1\top}\tilde{\boldsymbol{x}}^2),$$

we average it with respect to $\boldsymbol{J}$. Then, for any pair of two neurons in the same population, we calculate as

$$\frac{N}{2\pi g^2\sqrt{1-\eta_K^2}} \iint_{\mathbb{R}^2} \mathrm{d}\tilde{J}_{Ki,Kj}\mathrm{d}\tilde{J}_{Kj,Ki}\, \frac{g^2\sqrt{1-\eta_K^2}}{N} \exp\left\{ -\frac{g}{\sqrt{N}}\sqrt{1-\eta_K^2}(y^1_{Ki,Kj} + y^2_{Ki,Kj}) \right.$$

$$\left. J_{Ki,Kj} - \frac{g}{\sqrt{N}} J_{Kj,Ki}[(y^1_{Ki,Kj} + y^2_{Ki,Kj})\eta + (y^1_{Kj,Ki} + y^2_{Kj,Ki})] \right\} \exp\left[-\frac{1}{2}(J^2_{Ki,Kj} + J^2_{Kj,Ki}) \right]$$

$$\exp\left[-\frac{m_{KK}}{N_K}(y^1_{Ki,Kj} + y^2_{Ki,Kj} + y^1_{Kj,Ki} + y^2_{Kj,Ki}) \right]$$

$$= \exp\left\{ \frac{g^2}{2N}[(y^1_{Ki,Kj} + y^2_{Ki,Kj})^2 + (y^1_{Kj,Ki} + y^2_{Kj,Ki})^2 + 2(y^1_{Ki,Kj} + y^2_{Ki,Kj}) \right.$$

$$(y^1_{Kj,Ki}+y^2_{Kj,Ki})\eta_K] - \frac{m_{KK}}{N_K}(y^1_{Ki,Kj}+y^2_{Ki,Kj}+y^1_{Kj,Ki}+y^2_{Kj,Ki})\Bigg\}.$$

Additionally, for any pair of two neurons from different populations, we calculate as

$$\begin{aligned}
&\sqrt{\frac{N}{2\pi g^2}}\int_{\mathbb{R}} \mathrm{d}J_{Ki,Lj}\exp[-(y^1_{Ki,Lj}+y^2_{Ki,Lj})J_{Ki,Lj}]\ \exp\left[-\frac{N(J_{Ki,Lj}-m_{KL}/N_L)^2}{2g^2}\right]\\
&=\sqrt{\frac{N}{2\pi g^2}}\int_{\mathbb{R}} \mathrm{d}J_{Ki,Lj}\ \exp\left\{-\frac{N}{2g^2}\left[J_{Ki,Lj}+\frac{g^2}{N}\left(y^1_{Ki,Lj}+y^2_{Ki,Lj}\right)\right]^2\right\}\\
&\quad\exp\left[-\frac{m_{KL}}{N_L}(y^1_{Ki,Lj}+y^2_{Ki,Lj})+\frac{g^2}{2N}(y^1_{Ki,Lj}+y^2_{Ki,Lj}))\right]\\
&=\exp\left[-\frac{m_{KL}}{N_L}(y^1_{Ki,Lj}+y^2_{Ki,Lj})+\frac{g^2}{2N}(y^1_{Ki,Lj}+y^2_{Ki,Lj})^2\right].
\end{aligned}$$

Analogous to (SB5) computed in Appendix SB, we introduce the auxiliary fields and then obtain

$$\begin{aligned}
\bar{Z}\propto\int\prod_{\alpha=1}^{2}\left[\mathfrak{D}Q^\alpha\mathfrak{D}\tilde{Q}^\alpha\prod_{K\in\{E,I\}}\left(\mathfrak{D}R^\alpha_K\mathfrak{D}\tilde{R}^\alpha_K\mathfrak{D}T^\alpha_K\mathfrak{D}\tilde{T}^\alpha_K\right)\right]\mathfrak{D}U\mathfrak{D}\tilde{U}\exp\left[-\frac{N}{g^2}U^\top\tilde{U}\right.\\
-\sum_{\alpha=1}^{2}\frac{N}{g^2}Q^{\alpha\top}\tilde{Q}^\alpha+\sum_{K\in\{E,I\}}N_K\mathrm{ln}\upsilon_K+\sum_{K\in\{E,I\}}\sum_{\alpha=1}^{2}\left(-N_K R^{\alpha\top}_K\tilde{R}^\alpha_K-\frac{N}{g^2\eta_K}T^{\alpha\top}_K\tilde{T}^\alpha_K\right.\\
\left.+N_K\ln\omega^\alpha_K\right)+l_U^\top U+l_{\tilde{U}}^\top\tilde{U}+\sum_{\alpha=1}^{2}\left(l^\top_{Q^\alpha}Q^\alpha+l^\top_{\tilde{Q}^\alpha}\tilde{Q}^\alpha\right)+\sum_{K\in\{E,I\}}\sum_{\alpha=1}^{2}\left(l^\top_{R^\alpha_K}R^\alpha_K+l^\top_{\tilde{R}^\alpha_K}\tilde{R}^\alpha_K\right.\\
\left.\left.+l^\top_{T^\alpha_K}T^\alpha_K+l^\top_{\tilde{T}^\alpha_K}\tilde{T}^\alpha_K\right)\right],
\end{aligned}$$

where $U(t,t')=\frac{g^2}{N}\sum_{j=1}^{N}\phi(x^1_j(t))\phi(x^2_j(t'))$,

$$\begin{aligned}
\upsilon_K&=\prod_{\alpha=1}^{2}\left(\mathfrak{D}x^\alpha_K\int\mathfrak{D}\tilde{x}^\alpha_K\right)\exp\left[\tilde{x}^{1\top}_K(U+\sigma^2)\tilde{x}^2_K+\phi(x^1_K)^\top\tilde{U}\phi(x^2_K)\right],\\
\omega^\alpha_E&=\int\mathfrak{D}x^\alpha_E\int\mathfrak{D}\tilde{x}^\alpha_E\ \exp\left\{-m_{EE}\tilde{x}^{\alpha\top}_E R^\alpha_E-m_{EI}\tilde{x}^{\alpha\top}_E R^\alpha_I+\phi(x^\alpha_E)^\top\tilde{R}^\alpha_E+S[x^\alpha_E,\tilde{x}^\alpha_E]\right.\\
&\quad\left.+\frac{1}{2}\tilde{x}^{\alpha\top}_E Q\tilde{x}^\alpha_E+\phi(x^\alpha_E)^\top\tilde{Q}^\alpha\phi(x^\alpha_E)+\frac{1}{2}\tilde{x}^{\alpha\top}_E T^\alpha_E\phi(x^\alpha_E)+\phi(x^\alpha_E)^\top\tilde{T}^\alpha_E\tilde{x}^\alpha_E\right\},\\
\omega^\alpha_I&=\int\mathfrak{D}x^\alpha_I\int\mathfrak{D}\tilde{x}^\alpha_I\ \exp\left\{-m_{IE}\tilde{x}^{\alpha\top}_I R^\alpha_E-m_{II}\tilde{x}^{\alpha\top}_I R^\alpha_I+\phi(x^\alpha_I)^\top\tilde{R}^\alpha_I+S[x^\alpha_I,\tilde{x}^\alpha_I]\right.\\
&\quad\left.+\frac{1}{2}\tilde{x}^{\alpha\top}_I Q\tilde{x}^\alpha_I+\phi(x^\alpha_I)^\top\tilde{Q}^\alpha\phi(x^\alpha_I)+\frac{1}{2}\tilde{x}^{\alpha\top}_I T^\alpha_I\phi(x^\alpha_I)+\phi(x^\alpha_I)^\top\tilde{T}^\alpha_I\tilde{x}^\alpha_I\right\},
\end{aligned}$$

and the other notations are akin to (SB3) presented in Appendix SB. Then, we make a saddle point approximation and finally obtain

$$\begin{aligned}\bar{Z}^* \propto & \left\{ \prod_{\alpha=1}^{2} \left\{ \int \mathfrak{D}x_E^\alpha \int \mathfrak{D}\tilde{x}_E^\alpha \exp\left\{ S[x_E^\alpha, \tilde{x}_E^\alpha] + \frac{g^2}{2N} \tilde{x}_E^\alpha [N_E(1+\eta_E)\langle \phi(x_E^\alpha(t))\phi(x_E^\alpha(t'))\rangle \right.\right.\right. \\ & \left.\left. + N_I \langle \phi(x_I^\alpha(t))\phi(x_I^\alpha(t'))\rangle] \tilde{x}_E^\alpha - m_{EE}\tilde{x}_E^{\alpha\top}\langle \phi(x_E^\alpha(t))\rangle - m_{EI}\tilde{x}_E^{\alpha\top}\langle \phi(x_I^\alpha(t))\rangle \right\}\right\} \\ & \left. \exp\left\{ \tilde{x}_E^{1\top} \left\{ \frac{g^2}{N}[N_E(1+\eta_E)\langle \phi(x_E^1(t))\phi(x_E^2(t')) + N_I\langle \phi(x_I^1(t))\phi(x_I^2(t'))\rangle] + \sigma^2 \right\} \tilde{x}_E^2 \right\} \right\}^{N_E} \\ & \left\{ \prod_{\alpha=1}^{2} \left\{ \int \mathfrak{D}x_I^\alpha \int \mathfrak{D}\tilde{x}_I^\alpha \ \exp\left\{ S[x_I^\alpha, \tilde{x}_I^\alpha] + \frac{g^2}{2N} \tilde{x}_I^{\alpha\top} [N_E\langle \phi(x_E^\alpha(t))\phi(x_E^\alpha(t'))\rangle \right.\right.\right. \\ & \left.\left. + N_I(1+\eta_I)\langle \phi(x_I^\alpha(t))\phi(x_I^\alpha(t'))\rangle] \tilde{x}_I^\alpha - m_{IE}\tilde{x}_I^{\alpha\top}\langle \phi(x_E^\alpha(t))\rangle - m_{II}\tilde{x}_I^{\alpha\top}\langle \phi(x_I^\alpha(t))\rangle \right\}\right\} \\ & \left. \exp\left\{ \tilde{x}_I^{1\top} \left\{ \frac{g^2}{N}[N_E\langle \phi(x_E^1(t))\phi(x_E^2(t')) + N_I(1+\eta_I)\langle \phi(x_I^1(t))\phi(x_I^2(t'))\rangle] + \sigma^2 \right\} \tilde{x}_I^2 \right\} \right\}^{N_I}.\end{aligned}$$

Consequently, it is the moment-generating functional of the system

$$\begin{cases} \dfrac{\mathrm{d}x_E^\alpha}{\mathrm{d}t} = -x_E^\alpha + \gamma_E^\alpha(t) + \sigma\xi_E^\alpha(t) + m_{EE}\langle \phi(x_E^\alpha(t))\rangle + m_{EI}\langle \phi(x_I^\alpha(t))\rangle, \\ \dfrac{\mathrm{d}x_I^\alpha}{\mathrm{d}t} = -x_E^\alpha + \gamma_I^\alpha(t) + \sigma\xi_I^\alpha(t) + m_{IE}\langle \phi(x_E^\alpha(t))\rangle + m_{II}\langle \phi(x_I^\alpha(t))\rangle, \end{cases}$$

where each ξ_K^α with $\alpha = 1, 2$ and $K \in \{E, I\}$ is the standard white noise and each $\gamma_K^\alpha(t)$ with $\alpha = 1, 2$ and $K \in \{E, I\}$ is the stationary Gaussian process with the zero mean and the correlation satisfying

$$\begin{aligned}\langle \gamma_E^\alpha(t)\gamma_E^\beta(t')\rangle &= \frac{g^2}{N}[N_E(1+\eta_E)\langle \phi(x_E^\alpha(t))\phi(x_E^\beta(t'))\rangle + N_I\langle \phi(x_I^\alpha(t))\phi(x_I^\beta(t'))\rangle], \\ \langle \gamma_I^\alpha(t)\gamma_I^\beta(t')\rangle &= \frac{g^2}{N}[N_E\langle \phi(x_E^\alpha(t))\phi(x_E^\beta(t'))\rangle + N_I(1+\eta_I)\langle \phi(x_I^\alpha(t))\phi(x_I^\beta(t'))\rangle].\end{aligned}$$

This finally completes the validation.

SF. DYNAMIC EQUATION FOR DEFLECTION

Analogous to (SC1) presented in Appendix SC, we obtain

$$(\partial_t + 1)(\partial_{t'} + 1)\langle \delta x_K^\alpha(t)\delta x_K^\beta(t')\rangle = \langle \gamma_K^\alpha(t)\gamma_K^\beta(t')\rangle + \sigma^2\langle \xi_K(t)\xi_K(t')\rangle,\ \alpha, \beta = 1, 2,\ K \in \{E, I\},$$

that is,

$$\begin{aligned}(\partial_{t'} + 1)(\partial_t + 1)C_K^{\alpha\beta}(t, t') = & \frac{g^2}{N}[N_E(1+\delta_{KE}\eta_E)\langle \phi(x_E^\alpha(t))\phi(x_E^\beta(t'))\rangle) \\ & + N_I(1+\delta_{KI}\eta_I)\langle \phi(x_I^\alpha(t))\phi(x_I^\beta(t'))\rangle] + \sigma^2\delta(t - t').\end{aligned}$$

Notice that C_K^{11} and C_K^{22} satisfy (SC1) because they are the autocorrelation functions in the same dynamic. Thus, we only need to consider $C_K^{12} = C_K^{21}$. If it is a stable stationary solution, then it is consistent with C_K^{11} and C_K^{22}, which is trivial. Under the assumption of deflection (12), we make a Taylor expansion of $\langle \phi(x_K^1(t)\phi(x_K^2(t'))\rangle$ in the vicinity of ϵ and thus get

$$\begin{aligned}\langle \phi(x_K^1(t)\phi(x_K^2(t'))\rangle &= f_{\phi(\cdot+\langle x_K\rangle)}(C_K^{12}(t, t'), c_{K0}) \\ &\approx f_{\phi(\cdot+\langle x_K\rangle)}(C_K(t - t'), c_{K0}) + \epsilon f_{\phi'(\cdot+\langle x_K\rangle)}(C_K(t - t'), c_{K0})G_K(t, t').\end{aligned}$$

Since $C_K(t - t')$ is the solution of (SC1), the dynamic equation (13) as expected is obtained.

SG. PROOF OF PROPOSITION IV.1

Proof. When one of the conditions assumed in Proposition IV is satisfied, the dynamical equations of G_E and G_I are the same so that $G_E(t,t') = G_I(t,t')$. As $d_K(t) = -2\epsilon G_K(t,t)$, the maximal Lyapunov exponents for the two populations are identical. Letting $G_K(t,t') = H_K(t+t', t-t')$ and $H_K(T,\tau) = e^{\kappa T}\psi_K(\tau)$ leads to

$$-\partial_\tau^2\psi_K(\tau) - \frac{g^2}{N}\Big[N_E(1+\delta_{KE}\eta_E)f_{\phi'(\cdot+\langle x_E\rangle)}(C_E(\tau), c_{E0})\psi_E(\tau) \\ +N_I(1+\delta_{KI}\eta_I)f_{\phi'(\cdot+\langle x_I\rangle)}(C_I(\tau), c_{I0})\psi_I(\tau)\Big] = -(\kappa+1)^2\psi_K(\tau).$$

From the definition of the maximal Lyapunov exponent, it follows that $\lambda_{\max} = \kappa$. Since $G_E = G_I$, we thus conclude that

$$-\partial_\tau^2\psi_K(\tau) - \frac{g^2}{N}\Big[N_E(1+\delta_{KE}\eta_E)f_{\phi'(\cdot+\langle x_E\rangle)}(C_E(\tau), c_{E0}) \\ +N_I(1+\delta_{KI}\eta_I)f_{\phi'(\cdot+\langle x_I\rangle)}(C_I(\tau), c_{I0})\Big]\psi_K(\tau) = -(\kappa+1)^2\psi_K(\tau).$$

Together with V as defined in (7), $C_E = C_I$, $V_E = V_I$, and Price's Theorem, we obtain

$$-\partial_\tau^2\psi(\tau) + Y(\tau)\psi(\tau) = \left[1-(\kappa+1)^2\right]\psi(\tau), \tag{SG1}$$

where the subscripts are omitted for simplicity, $Y(\tau) = -X''(C(\tau))$, and $X(C) = V(C,C)$. It is the form of the Schrödinger equation [34]. In light of the Sturm-Liouville theory, Eq. (SG1) possesses countable solutions satisfying $\psi(\infty) = 0$. It can be easily verified from (8) and (SD1) that, $|C'(\tau)|$, having no zero point, is a well-posed solution of Eq. (SG1). Here, the well-poseness of a solution is assured if $C''(0) = 0$, and such a solution corresponds to the ground-state energy $E_0 = 1-(\kappa_0+1)^2$ [39]. Also, it follows from the Node Theorem [17] that its associated eigenvalue is the maximal one of Eq. (SG1). In our case, this eigenvalue is uniquely attained as $\kappa_0 = 0$. As a consequence, chaotic behaviour occurs since the MLEs of both populations are zero. To guarantee the validity of $C''(0) = 0$, the following equation needs to be satisfied

$$c_{K0} - \frac{g_{K,c}^2 N_E}{N}(1+\delta_{KE}\eta_E)f_{\phi(\cdot+\langle x_E\rangle)}(c_{K0}, c_{K0}) - \frac{g_{K,c}^2 N_I}{N}(1+\delta_{KI}\eta_I)f_{\phi(\cdot+\langle x_I\rangle)}(c_{K0}, c_{K0}) = 0.$$

Specifically, the critical point $g_{K,c}$ satisfies

$$c_{K0} - g_{K,c}^2\left(1+\frac{N_K\eta_K}{N}\right)f_\phi(c_{K0}, c_{K0}) = 0,$$

if we choose an odd transfer function (for instance, arctangent function used in this work) or if the means of the two populations vanish.
